# Analysis of Bonding by Quantum Chemistry—Resolving Delocalization Stabilization in a Mechanistic Basis and New Hückel Model

**DOI:** 10.1021/acs.jpca.2c08497

**Published:** 2023-04-11

**Authors:** Sture Nordholm

**Affiliations:** Department of Chemistry and Molecular Biology, The University of Gothenburg, 412 96 Gothenburg, Sweden

## Abstract

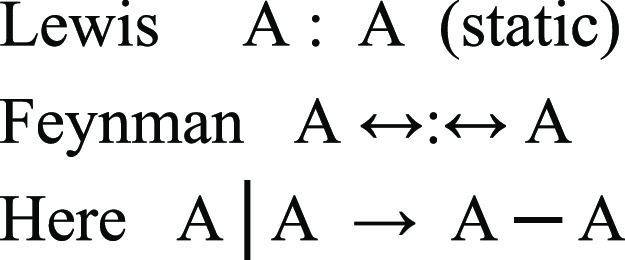

No general and unique understanding of the mechanism of covalent bonding in physical terms is provided by current computational methods or by a consensus among experts. Bonding is studied by energy decomposition analysis but may also be related to the interatomic motion of valence electrons within the molecule. This dynamical view of the mechanism of bonding is not widely appreciated. The aim here is to make it accessible by translation into a corresponding form of quantum chemical energy analysis. The interatomic electron motion is directly related to the delocalization taking place when atomic basis functions are combined into molecular orbitals. A “tribasis method” is introduced, allowing an atomic basis set to form subsets of (1) strictly localized atomic functions and (2) interatomic bridge functions which allow delocalization. Calculations can then identify ground states without (no bridge functions) and with delocalization. The scheme is based on exact quantum mechanics but demonstrated by a minimal basis treatment of H_2_^+^ and H_2_ in Hartree–Fock and valence bond approximations which show that the bond energy is a sum of repulsive localization and more strongly attractive delocalization energies. The tribasis method is used to reconstruct the Hückel theory of π-electron delocalization in planar hydrocarbon molecules to account for the “overlap problem”. In its empirically fitted form, the new theory can accurately resolve both π → π* transition energy and aromatic stabilization energy. The picture of covalent bonding emerging from both hydrogenic and Hückel calculations is that there is a presence of a Pauli repulsion of localization which is overcome by a roughly twice as strong delocalization stabilization to form the bond.

## 1 Introduction

The remarkable development of quantum chemistry, and ever more powerful computers, has made it possible to resolve the electronic structure of molecules and the effects of chemical bonding to great accuracy, but the computational methods available for the purpose do not come with physical explanations of bonding mechanisms. The result is that there are still, among experts and chemists and physicists in general, diverse, and sometimes conflicting, opinions about the origin of the bonding. This is particularly true with respect to covalent bonding, whereas ionic bonding involving electron transfer from one atom to another is less controversial. A likely source of this uncertainty about the mechanism of covalent bonding is the fact that it is very fundamentally a quantum mechanical phenomenon. The fact that the search for a quantum ground state involves the minimization of the sum of kinetic and potential energies has led to a protracted debate about which of these types of energies should be considered the source of the covalent bond. A major division has arisen between adherents of the electrostatic interactions (Slater 1933, Feynman 1939)^2,3^ and the kinetic energy (Hellmann 1933, Ruedenberg 1962)^4,5^ as the key to the bonding. Many quantum chemists have accepted the Hellmann–Ruedenberg view that the lowering of kinetic energy associated with delocalization of electrons over two or more atomic centers in a molecule is the key to the bonding. Nevertheless, the apparent conflict with the virial theorem and the simultaneous localization of electron density around the atomic nuclei as the molecule approaches its equilibrium geometry need to be understood before this view can be seen to agree with calculations. Thus, the resolution of the role of “orbital contraction”, as reflected in the virial theorem, has been decisive in establishing the Hellmann–Ruedenberg view of covalent bonding among quantum chemists,^5−8^ but the debate continues. Apart from delocalization and orbital contraction, much focus is on the further subdivision of the energy by decomposition analyses into terms associated with what is normally referred to as electrostatic interaction, delocalization, and Pauli repulsion.^9−16^ Unfortunately, no uniquely fundamental, or clearly superior, way to do this decomposition has yet appeared.

**Table 1:** Carbon–Carbon Bond Strengths in kJ/mol

bond strength	average energy/kJ/mol^a^
C–C (σ)	348
C=C (σπ)	612
C≡C (σπ^2^)	838
aromatic C–C (σ and delocalized π)	518

a Enthalpies are given, but the corrections due to nuclear motion are small, and we compare the same number of bonds in the difference below.

In quantum chemistry, one nearly exclusively studies the spectra of energy eigenstates, with particular focus on ground states, of atoms and molecules with calculations sometimes extended to excited states. Thus, one studies “stationary states”, i.e., apparently time-independent properties of the system. However, the non-vanishing kinetic energy tells us that the electrons are moving, and the nature of the motion is reflected in the geometry of the wave function and in its energy.^17^ This strong coupling between electron dynamics and the shape and energy of their ground and excited states is a hitherto somewhat neglected fact in the study of chemical bonding. It is possible that the covalent bonding mechanism is more readily identified by its dynamical character (e.g., localized or delocalized electron motion) than by the decomposition of the ground state energy into contributions of variable kinetic and potential energy.

The possibility and advantages of a dynamical description of covalent bonding were noticed and promoted by Feynman^18^ who in his famous Feynman Lectures on Physics described covalent bonding as the result of a “flip–flop” motion of valence electrons between atomic sites in molecules. He made no mention of the implied contradiction of his earlier view^3^ that the bonding was an electrostatic phenomenon. Similarly, Ruedenberg^5^ was aware of this possibility of a dynamical description of bonding but did not consider it advantageous. This may be seen as an illustration of the fact that many features of the formation of bonds and molecules from atoms can be suggested as the “mechanism of bonding”. The best, or most correct, mechanism must be chosen on the grounds of its generality, clarity, and utility. Moreover, preference must be given to a mechanism which is not only a contributor to bonding but also essential to it. This criterion speaks, we believe, in favor of interatomic electron dynamics and delocalization of wave functions over two or more atomic centers but against the contractive electrostatics of the virial theorem as key features of covalent bonding.^19^

The difficulty of resolving reactivity and bonding in semi-classical density functional theory (DFT) of electronic structures, e.g., Thomas–Fermi theory, and ease of doing so in pure quantum mechanics, e.g., Hückel theory, have long been convincing arguments for the generality and utility of the dynamical mechanism of covalent bonding,^20,21^ but this remains very much a minority view. The reason may be that the close coupling between dynamics and stationary energy eigenstates in quantum mechanics is not widely known. Thus, our work presented below is devoted to the task of connecting calculations of stationary quantum ground and excited states to the underlying dynamics of the valence electrons that we all know to be the agents of bonding. Our claim is that in dynamical terms, “covalent bonding results from stabilization due to the interatomic motion of valence electrons”. We regard this view of the mechanism of covalent bonding to be completely consistent with the Hellmann–Ruedenberg picture of bonding. The advantage is that the dynamical view is more general. It is not exposed to the subtleties of energy decomposition analysis relating to the simultaneous presence of diffusive interatomic and contractive intra-atomic changes of the electronic wave function and density. Our task here is to clarify the relation among the quantum chemically calculated wave function, density, and energy, as employed in energy analysis of bonding, and either the local atomic or interatomic nature of the electron motion.

The phenomenon of interatomic electron motion is, we claim, already established in quantum chemistry but under the name “electron delocalization” and related to ground state wave function character and energy.^10−16^ Other terms are also used for essentially the same mechanism, e.g., constructive interference of atomic orbitals, resonance, or just covalent contribution. The underlying mechanism is the same but seen in the wave function picture as a formation of a molecular orbital or state from atomic orbitals or configurations. The same underlying quantum mechanical reality is captured in a range of words and concepts with uncertainties related to the “basis function choice”-driven nature of the computational methods.

The atomic basis sets which very efficiently resolve both the atomic structure and chemical bonding in molecules do not, in the usual manner of use, precisely define the process of delocalization. The reason is that these atomic basis functions overlap in the formation of the molecule and therefore become partially delocalized even before the molecular wave function is formed from them. Thus, it is difficult to extract the equivalent electron dynamics from the usual quantum chemical methodology which is optimized for efficient representation of delocalization but cannot readily represent the local initial state needed to determine the full effect of delocalization. We therefore propose here a type of basis set reconstruction which turns a traditional basis set of atomic orbitals into a set of localized atomic orbitals, with no overlap if they belong to different atoms, and a subset of bridge basis functions which precisely reintroduce the needed coupling between the local atomic subspaces. In this way, we can now rigorously define local ground states of molecules with no interatomic electron motion. This is done simply by removing the bridge states from the basis. When we then reintroduce the bridge states, the delocalized ground state can be obtained with a precisely identifiable stabilization assigned to “dynamical delocalization”, i.e., to the motion of electrons between atomic subspaces.

The basis set we introduce here for homogeneous molecular structures will be called the “tribasis” because it will be used to produce a set of three basis functions from two identical atomic orbital functions on neighboring atoms. This is done by first creating the ground and first excited diatomic orbitals from the two atomic orbitals and then extracting two local atomic orbitals from the first excited (antibonding) diatomic orbital by cutting it at its node in the bond mid-plane. The joint space formed by the initial two atomic basis functions and the two local atomic basis functions is three-dimensional since the delocalized excited state is just the subtraction of the two local atomic states. Three orthogonal basis functions spanning the space can be formed by projecting the two local states out of the symmetric combination (bonding) of the initial atomic basis functions. The delocalized state so formed will be the bridge function which allows electrons to move between the two atomic half-spaces. It is important to note that the same tribasis construction can start from the exact ground and first excited states of a one-electron double well problem. The result will then be a basis set of two local atomic energy eigenstates and a bridge function allowing the construction of the exact ground and first excited delocalized states.

For small molecules, we use H_2_^+^ and H_2_ as examples; a good description of covalent bonding can be obtained in a basis of only two atomic orbitals, the minimal basis. Thus, we illustrate the new basis method by reconstructing this minimal basis into a “tribasis” of two local and one bridge functions. The 3D function space obtained contains the ground and first excited state wave functions of the minimal basis but also two strictly local atomic basis functions which are also local energy eigenstates. The functionality is therefore essentially complete for our purpose of studying delocalization stabilization. We then show how the presence or absence of the bridge function produces global and local ground states which differ in energy by the “dynamical delocalization energy” directly related to interatomic electron motion. The total energy obtained by the tribasis is just slightly better than that obtained in the minimal basis itself for H_2_^+^ and for H_2_ in the Hartree–Fock approximation. The bond energy is nearly precisely the same in the valence bond (VB) treatment of H_2_. The tribasis is not effective in improving the bond energy. It is an analytical device designed to resolve the origin of covalent bonding. It allows the dynamical delocalization energy to be calculated by quantum chemistry and shows that it is considerably greater than the bond energy since the local state, from which it is initiated, is of higher energy than the atomic ground state due to a “Pauli repulsion of localization”. Interestingly, the Hartree–Fock ground state of H_2_ in the full tribasis is, according to our calculations mentioned below, only delocalized for bond lengths *R* < 4 au. For larger bond lengths, the local singlet state is lower in energy and approaches the correct separate atom limit. The more accurate VB treatment of H_2_, on the other hand, is hardly affected by the tribasis reconstruction. This illustrates an important fact of the quantum chemistry of bonding: the assumption of fully independent electron motion fails in the large bond length limit where correlation is needed to resolve the bonding. Nevertheless, the dynamical delocalization is still the mechanism of bonding. If we form the ground state from only the two VB configurations of local atomic character, which minimize electron–electron repulsion, the bond energy is repulsive. The bond energy only becomes attractive when configurations involving bridge states are added to allow electrons to move between atoms.

Hückel molecular orbital (HMO) theory^22^ has long been used to understand the π-bonding in planar hydrocarbon molecules.^23^ It is of particular interest here for two reasons: (i) it is a theory of delocalization which extends a parameterization from two atoms to many atoms connected in chains or rings, thereby allowing study of collective features of bonding like aromaticity; (ii) the connection between orbital delocalization and similarly delocalized electron motion has been emphasized. We have noted the strength of HMO theory as a tool of bonding analysis.^24^ The drawback of the original HMO model is mainly to be found in its unrealistic neglect of overlap and non-orthogonality among the atomic basis functions used. If we apply the tribasis method to the diatomic Hückel structure of ethylene, we have two rigorously local π-basis states of atomic character and one bridge function which ensures that delocalization can be resolved. We therefore take our tribasis construction and apply it to generalize Hückel theory so that it can, by quantum chemical methods, be more clearly related to its foundation of one-electron theory of bonding. The resulting theory is, by the representation of both atomic sites and interatomic bridges, of higher dimensionality than the original HMO model but still amenable to analytical solution for many important structures. The new tHMO model is reliant on three parameters which can be either derived or determined empirically. We show that the parameters can be chosen so that the bond energy and π → π* transition energy of ethylene and the aromaticity of benzene are accurately reproduced.

## 2 Theory—The Definition of Delocalization

The great progress of quantum chemistry in the last 100 years has been due in large measure to the successful use of atomic basis functions to solve the Schrödinger equation for the canonical orbitals and ground state wave functions of molecules. With respect to the molecule as the global system, the atoms in it are local sites, and the atomic orbitals are in this sense local basis functions used to generate global energy eigenfunctions of the molecule. If this is accurately achieved for the ground state wave function, then delocalization has been accomplished with respect to bonding in the molecule. There is no question that successful representation of the global nature of the electronic ground state is an essential requirement of a theory of covalent bonding, but there is a question about how much of the stability of the molecule should be assigned to “delocalization”. The answer is needed for its contribution to be compared with that of other mechanisms, most particularly in an energy analysis of bonding.

**Figure 1:**
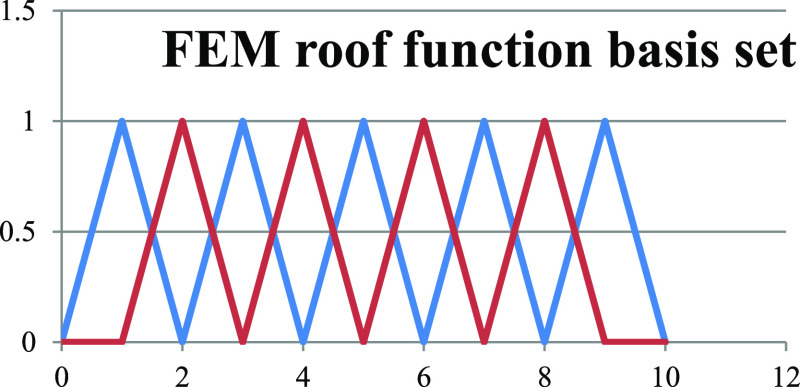
Well-coupled FEM roof function basis set for the interval 0 < *x* < 10.

The problem of making local basis functions accurately resolve global solutions of some differential equation is of general interest in numerical analysis. A famous illustration of how this problem can be solved is provided by the finite element method (FEM).^25^ We use this method in its simplest form to illustrate the key idea of this paper. The simplest FEM basis function in one dimension (1D) is the “roof function”. A basis set known to effectively reproduce global functions obtained, e.g., as solutions of a differential equation, is a string of overlapping roof functions as shown in Figure 1.

Naturally, the spacing of these roof functions controls the refinement with which a global function can be reproduced. The suitability of the spacing as shown, i.e., half the width of the roof function, is indicated by the fact that this basis set produces a precise piecewise linear fit of a global function. We have previously noted the numerical similarity between translation described by the roof functions and delocalization described by the Hückel Hamiltonian.^24^ Another point of interest is that if we use such a basis in the solution of the Schrödinger equation in 1D, it is easy to introduce localization of particle motion. If we remove the roof function centered on *x* = 5, then this forces the solutions to a boundary condition ψ(5) = 0, and the energy eigenfunctions will behave as if there is an infinitely thin but impenetrable wall at *x* = 5. The solutions to the Schrödinger equation will then be left- or right-localized with respect to the point *x* = 5, and so will the dynamics of the particle. The roof function centered at *x* = 5 is a “bridge function” since without it, we have two separate domains which are fully joined when the bridge function is restored to the basis set. We have found a convenient off–on switch in the basis set used to describe localized and delocalized forms of particle motion.

**Figure 2:**
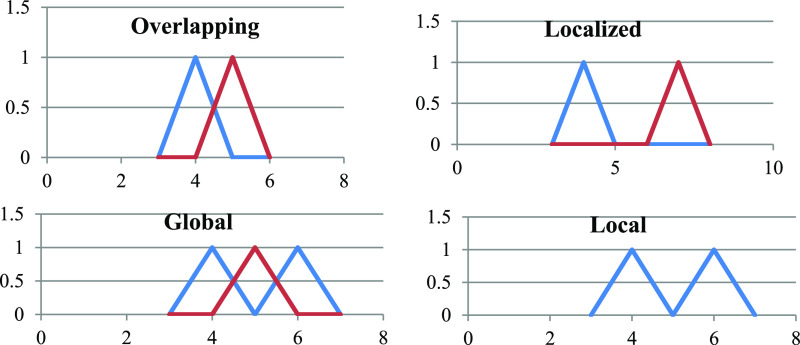
In the top left graph, we show two roof basis functions positioned with optimal overlap to describe delocalization over the interval 3 < *x* < 6. On the top right, they have moved apart to eliminate overlap and thereby also ability to resolve left–right coupling and delocalization. In the lower graphs, we illustrate the key idea of the tribasis method. On the left, a set of three neighboring FEM functions are optimally able to describe delocalization over the interval 3 < *x* < 7, but it becomes on the right a set of two local basis functions on the same domain without left–right delocalization by removal of the bridge function between them.

We see from the role of the bridge roof function that overlap of local basis functions facilitates delocalization. If the overlap is eliminated at a point by removal of the roof function centered on this point, then particle motion will be localized to either side of that point. Similarly, basis functions with subsets physically separated from each other cannot describe motion between their respective domains. This follows from the fact that the kinetic energy operator cannot then couple basis functions across this separation. The role of overlap is illustrated in a cartoon-like manner in Figure 2.

The attraction of local basis functions is that they can, in an obvious extension of the simple FEM mentioned above, be adapted to local features of the underlying potential and particle motion. Local basis functions may then be chosen to be local eigenfunctions of the Hamiltonian so that the global basis set becomes a collection of local energy eigenfunctions. This idea has been explored in the generalized FEM^26,27^ which is clearly related to the atomic basis set schemes of quantum chemistry. Indeed, this “efficiency of local but overlapping energy eigenfunctions” is the great advantage of atomic basis functions in quantum chemistry, leading to smaller, and computationally economical, basis sets. An additional advantage is that atomic orbitals make it possible to physically interpret the computational results as amply illustrated in VB and linear combination of atomic orbital–molecular orbital models of molecules.^28^

For symmetric double-well potentials, a particularly useful “local plus bridge basis set” is available. We note that the energy eigenfunctions in order of increasing energy ψ_0_(*x*), ψ_1_(*x*), ψ_2_(*x*), ... are even and odd functions around the midpoint of the potential. Thus, the odd-numbered eigenfunctions are also odd with respect to symmetry around the midpoint, and they can therefore, by a cut at the midpoint where they vanish, generate local eigenfunctions of the left and right halves of the axis and potential. In this way, the ground and first excited states of a double-well potential generate a very effective tribasis of two local ground states and one global ground state which can easily be orthogonalized to the former. The global orthogonal function thereby contributes a bridge basis function centered on the bond midpoint in a basis set of three functions which optimally describe the process of delocalization at low energy in the double well. Using only the two local functions, we get the degenerate ground states of the double well under the condition that there is no particle motion between the wells. Adding the bridge function then allows us to get the exact delocalized ground state and first excited state. The decrease in energy associated with the addition of the bridge basis function then becomes our “delocalization stabilization”. This definition precisely defines the “local initial state” to which delocalization is applied, and the definition only involves one system of a given Hamiltonian with or without a localization constraint. The basis set so obtained will be referred to as “the tribasis”.

## 3 Results—Dynamic Delocalization in H_2_^+^ and H_2_ by the Tribasis Method

### 3.1 H_2_^+^

The tribasis scheme introduced above readily generalizes from one- to three-dimensional particle motion in an axially symmetric double-well potential as presented by a diatomic molecule such as H_2_^+^. The reflection symmetry dictates that localization of the particle motion shall be implemented by introduction of an impenetrable plane orthogonal to the bond axis (the *x*-axis) and passing through the bond midpoint. The delocalization energy can now be determined for any bond length *R* from the set of left, right, and global eigenfunctions ψ_l,0_, ψ_r,0_, and ψ_0_ and their corresponding energies ε_l,0_, ε_r,0,_ and ε_0_. Here, the left- and right-localized energies are identical by reflection symmetry and the latter global energy is lower than the local energy. From the fact that the local ground states are lobes of the global first excited state, we propose that the “dynamic delocalization energy”, Δ*E*_del_ (defined as negative like a bond energy), be defined as
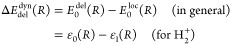
1

Here and below, we work in the Born–Oppenheimer (BO) approximation of infinite atomic masses. Capital *E* will refer to total BO system energies, including any nuclear repulsion present, while ε will refer to electronic energies in the nonrelativistic approximation without nuclear repulsions and spin–orbit or spin–spin interactions. *E*_0_^del^ and *E*_0_^loc^ mentioned above are the delocalized and localized ground state energies, respectively, and ε_0_ and ε_1_ are the electronic energies of the bonding and antibonding one-electron energy eigenstates, respectively.

This means that the delocalization energy Δ*E*_del_^dyn^(*R*) is the energy difference between the delocalized ground state and the corresponding local ground state at a given bond length *R*. In the case of H_2_^+^, with just one electron moving in a symmetric potential, the lowering of energy associated with release of the constraint (that the electron moves only in its original half-space) is the separation between the lowest and next lowest orbital energies in the spectrum of the electron motion around the stationary nuclei. Thus, the delocalization energy is precisely the (negative) gap between the lowest pair of antibonding and bonding molecular orbitals.

It is possible to do the one-electron calculations to determine the “dynamic delocalization energy”, defined above, exactly, but we shall be satisfied here with a simple numerical exploration of this concept. A good result for the ground state properties of H_2_^+^ can be obtained already by the minimal basis of two H 1s atomic orbitals centered at *x* = ±*R*/2 along the bond axis and here denoted φ_1s,a_ and φ_1s,b_. As is well known, the sum of these two functions is a good approximation for the ground state and the difference between them for the first excited state
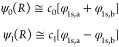
2

**Figure 3:**
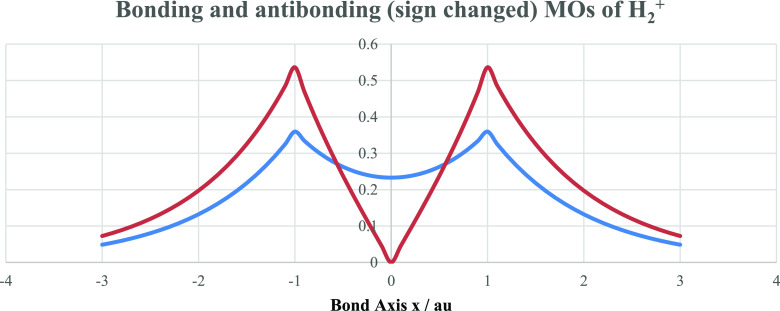
Bonding and antibonding (with sign change for *x* > 0) orbitals for H_2_^+^ in the minimal basis of H 1s functions and atomic units. Plots here and given below show a truncated view.

**Figure 4:**
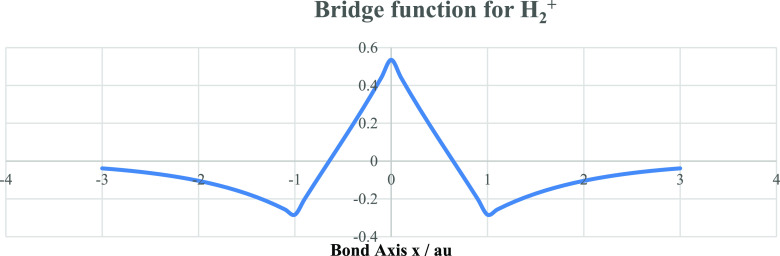
The bridge function (in au) is shown as obtained from the minimal basis of H 1s atomic orbitals.

From these two eigenfunctions, i.e., molecular orbitals (MOs) (see Figure 3), we now construct the tribasis by cutting ψ_1_ into two separate lobes, left and right of the bond mid-plane, and retaining ψ_0_ which is then orthogonalized to the two local basis functions. In this way, ψ_0_ generates a bridge function (see Figure 4) which couples the local basis functions allowing them to resolve delocalization equally well as in the original minimal basis set. In fact, the tribasis can resolve the delocalization (a little) better than the original minimal basis because the amount of the bridge function in the ground state can be optimized, while in the original minimal basis, the bridge function is present with a fixed weight related to the overlap of the atomic basis functions. As it turns out, the minimal basis with its long-range exponential fall off produces good overlap and resolves delocalization very well, but this is to a degree a lucky circumstance. If we were to use small Gaussian expansions instead of full exponentials in the H 1s orbitals, we might see deteriorating bridging and greater need for the optimization offered by the tribasis.

**Figure 5:**
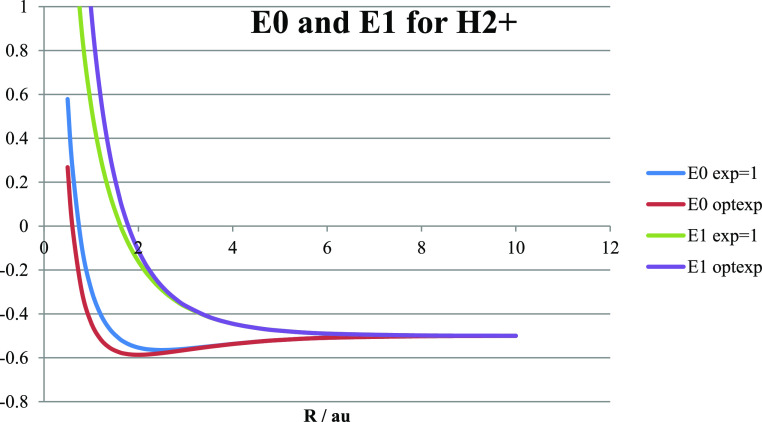
Ground and first excited state energies in hartree (= 2625.5 kJ/mol) of H_2_^+^ (truncated view) as obtained in the minimal tribasis with the exponent in the H 1s basis functions kept fixed at the atomic value ζ = 1 (inner curves) or optimized (lowest and highest).

The calculations of bond energy Δ*E*_b_(*R*) for H_2_^+^ can be done with the exponent ζ in the basis frozen at its value for the hydrogen atom, ζ = 1, or it can be optimized for each bond length. The latter yields a rise in the exponent with decreasing *R* which becomes significant close to the equilibrium at *R*_e_. The equilibrium bond length *R*_e_ becomes a little shorter and the bond strength a little greater by this exponent optimization, but it leaves the tribasis construction unchanged, and the stabilization by bridge contribution optimization in the tribasis is small, i.e., no more than about 20 kJ/mol compared with a (vibrationless) bond strength of about 240 kJ/mol at *R*_e_. The ground and first excited state energies *E*_0_ and *E*_1_ of H_2_^+^ in the “minimal tribasis” are shown in Figure 5, with and without exponent optimization to generate the lowest ground state energy. Note that the inner two curves correspond to the calculations in the original ζ = 1 basis which produce lower binding energy but also lower antibonding repulsion in the first excited state which has the same energy as the local ground state. Thus, we see that the exponent optimization for the delocalized ground state is ineffective (*R* > *R*_e_) or non-optimal (*R* ≤ *R*_e_) for the local BO ground states and the first excited delocalized state. In our deliberations here, *R*_e_ will be the bond length of minimal total BO energy. Shifts in *R*_e_ due to zero-point vibrations are very small for the systems considered and unimportant for our bonding analysis.

**Figure 6:**
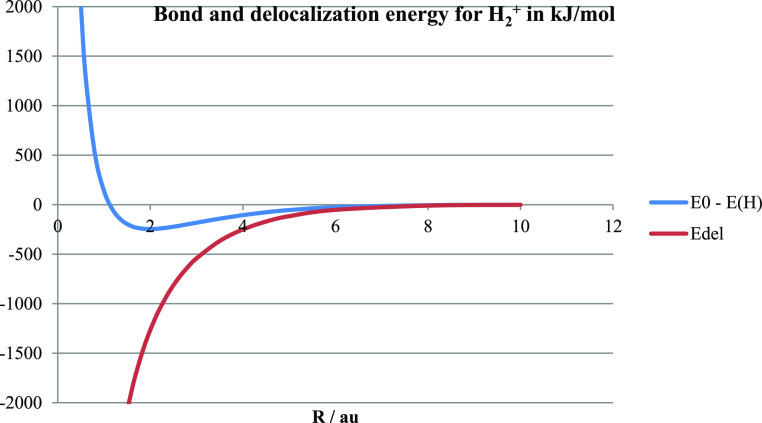
The bond and delocalization energies (kJ/mol) of H_2_^+^ are shown as obtained in the minimal tribasis method with the optimized exponent in the 1s basis functions.

The bond and delocalization energies of H_2_^+^ in the minimal tribasis with optimized exponent ζ in the atomic basis functions are shown in Figure 6, where the bond energy is simply the ground state energy of H_2_^+^ relative to the energy *E*(H) of a H atom (1/2 hartree), i.e.,
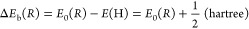
3

We find that the magnitude of the dynamical delocalization energy is quite large. For relatively large *R* (>4 au), it is about twice as large as the electronic part of the bond energy which is normally taken to be the ground state orbital energy ε_0_ minus its value at infinite separation, ε_0_(*R*) – ε_0_(∞) = ε_0_(*R*) + 1/2 in au. The coupling of the basis functions in the unrestricted and delocalized form of the calculation leads, as seen in Figure 5, to a splitting of the two molecular orbitals so that the average energy of the ground (bonding) and first excited (antibonding) states is initially almost constant, while the orbital energies ε_0_(*R*) and ε_1_(*R*) move down and up in roughly equal measure with decreasing *R*. Thus, we find the bond energy expressed traditionally and as proposed here in two different ways [*E*(H) is the energy of the hydrogen atom, i.e., −1/2 au]
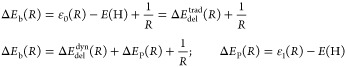
4

Note that in eq 4, we assign all the bond energy to “traditional delocalization energy” on the basis that previously, there has been no truly local state available to compare with for an arbitrary bond length *R*. The general notion has been that an atomic H 1s orbital has been localized on its atom of origin, but as emphasized here, such an orbital has a presence around also the “other proton” in H_2_^+^ for any finite *R*. Only in the case of infinitely large *R* can we say that the H 1s orbital is localized on its atom of origin in H_2_^+^. For this reason, it makes sense to say that “traditional delocalization” must be defined in comparison between the energy of a delocalized H_2_^+^ and the energy in the separated atom limit.

The dynamical delocalization energy Δ*E*_del_^dyn^(*R*) is a difference in energy between global and local ground states for a given *R*, while the traditional delocalization energy is a change in delocalized ground state energy as *R* goes from infinity to some finite value. A new repulsive term Δ*E*_P_(*R*), the rise in energy with decreasing *R* of the local ground state, enters the expression for the bond energy in terms of the dynamical delocalization energy. This is a type of Pauli repulsion energy associated with the overlap of the H 1s basis functions which is eliminated in the two local ground states. It is related, but not equivalent, to the usual Pauli repulsion associated with the orthogonalization that is normally carried out for an atomic basis set. The latter results in overlapping but orthogonal functions, but for rigorously localized electron motion, no overlap is allowed. In the tribasis, the lobes of the first excited delocalized orbital are both orthogonal and without overlap. In the case of H_2_^+^, the level splitting is nearly symmetric around the asymptotic H 1s value −1/2 au. We have in au (hartree)
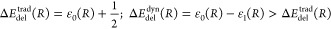
5

For not too small bond lengths (*R* > 4 au say), the dynamical delocalization energy is, as noted, roughly twice the traditional one. Given that the bond energy is nearly the same in the tribasis as that in the minimal basis, this means that the Pauli repulsion of localization is roughly as large as the traditional and half as large as the dynamical delocalization energy but of the opposite sign, i.e.,
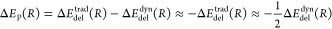
6

A reservation related to the use of a minimal basis set of 1s type atomic basis functions should be added here. The tribasis analysis should in principle be carried out using exact eigenfunctions of the H_2_^+^ molecule. Our simplified exploration of the tribasis results is based on the acceptable accuracy of the minimal basis for the ground state and corresponding bonding energy, particularly when the exponent ζ of the exponential function is scaled to account for the orbital contraction setting in as the molecule approaches its united atom form as He^+^. This scaling is not equally applicable to the local ground state of H_2_^+^ which in the same united atom limit approaches a form of two lobes either side of a plane containing a helium nucleus. The lobes are more diffuse than allowed by the minimal basis construction since they correspond at *R* = 0 to the 2p orbital of He^+^. For this reason, the local ground state will not be accurately reproduced for bond lengths *R* small enough to show significant scaling of ζ. Thus, the delocalization and Pauli-like localization energies will not be reliable for *R* < 2 au. This is a numerical limitation that we accept for the moment. The bond energies produced by the tribasis are still good, and the interpretations offered in our analysis remain qualitatively correct, if numerically exaggerated for small *R*.

The tribasis analysis has brought out a Pauli repulsion mechanism associated with localization of electron motion, which is always present when there is covalent bonding, even for H_2_^+^, if we are going to identify a delocalization mechanism starting from localization. It is a major mechanism roughly as strong as the bonding itself. The recognition of this mechanism leads also to a reevaluation of the delocalization stabilization which is found to be roughly twice stronger than previously estimated. Note also that the concept of dynamical delocalization is clearly related to allowing the electron to cross the bond mid-plane and oscillate between atomic centers. On the other hand, the traditional method of combining atomic orbitals into delocalized molecular orbitals does not have a simple dynamical interpretation given that atomic orbitals already overlap and display partial delocalization. In the united atom limit, the atomic H 1s orbitals we use here become ground states of He^+^ and therefore completely delocalized. The tribasis analysis of dynamical delocalization starts from an initial state which is local for all bond lengths and approaches He 2p character in the united atom limit.

**Figure 7:**
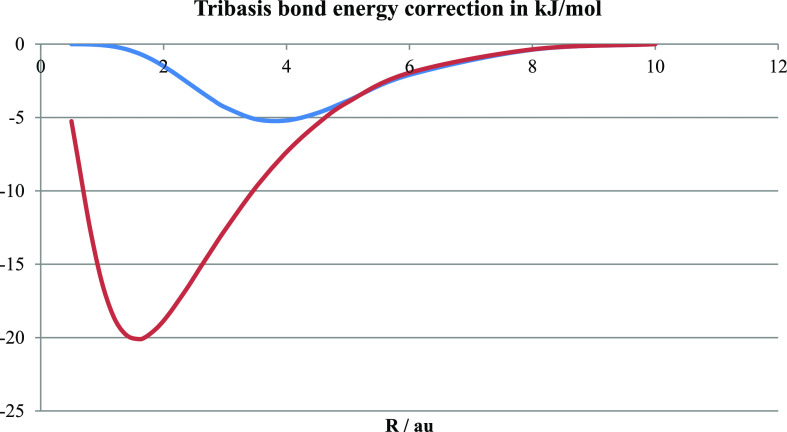
Shown here is the improvement in bond energy of H_2_^+^ due to variational optimization of bridge function contribution rather than using the overlap-determined contribution of the original minimal basis. The lower curve is for the optimized 1s exponent, while the top curve is for the original (fixed) H 1s exponent of 1.0.

The purpose of the tribasis is to allow a precise definition of delocalization energy with a clear connection to electron dynamics, but there is also an advantage in that the contribution of the bridge function can be determined variationally rather than being prescribed by the original atomic basis as in the case of the minimal basis calculation for H_2_^+^. This should lead to better ground state and bond energies. It does, but the effect is small as seen in Figure 7.

We see that the tribasis correction of the bond energy relative to that of the minimal basis is no more than 5 kJ/mol around *R* = 4 au for the calculation with the original H 1s basis, while the exponent optimization increases it to a maximum of 20 kJ/mol at about 2 au. Thus, the bond energies obtained with the original minimal basis are very marginally improved by the tribasis reconstruction which is designed to aid bonding analysis rather than improve the related energies. The larger effect seen for the optimized atomic basis is presumably due to the withdrawal of electron density from the bridge caused by the contraction around the nuclei effected by the larger exponent ζ. This is counteracted by an increase in the contribution of the bridge basis function.

#### 3.1.1 Implication for the Bonding in H_2_^+^

We conclude from the above that the traditional minimal basis of overlapping 1s atomic basis functions resolves delocalization in H_2_^+^ very nearly as well (to within 10%) as the more flexible tribasis. The major gain achieved by the tribasis is that it allows us to define and determine the energy of the local ground state of H_2_^+^. Thus, we can now determine the energy difference between local and delocalized ground states and thereby obtain a delocalization energy properly associated with interatomic electron motion. This dynamical delocalization energy is the full energy separation between the lowest pair of antibonding and bonding of H_2_^+^ and roughly twice as large as the bond energy. This large energy difference arises due to the presence of a strong “localization repulsion” related to the prevention of electron motion between atomic half-spaces. Thus, rigorous analysis of one-electron covalent bonding brings in Pauli-like repulsion opposing a stronger attraction due to interatomic electron motion.

### 3.2 H_2_—Independent or Correlated Motion?

The addition of an electron to create H_2_ from H_2_^+^ brings important changes, in particular due to electron–electron interaction and the presence of correlation which requires introduction of approximation, but the basis sets used above still apply. We shall see here what results they produce within the mainstream approaches to the electronic structure, Hartree–Fock and VB theory,^28^ and consider the implications of these results for the determination of delocalization energy and its role in the covalent bond. These two theories make nearly opposite assumptions about the motion of the two electrons: This simple MO and, in general, the Hartree–Fock MO theories assume that the two electrons move independently, while VB theory assumes that their motion between the atomic centers is nearly perfectly correlated so that two electrons are never (almost) at the same atomic center. It is well known that the independent (uncorrelated) motion assumed in Hartree–Fock theory normally works well around *R*_e_ but fails for large *R*. Nevertheless, the structure of the theory in terms of one-electron molecular orbitals occupied by 0, 1, or 2 electrons is widely used for molecular systems, e.g., in modern DFT^29,30^ where the correlation effects are treated by refined semi-empirical approximations.

The form of the Hartree–Fock wave function for H_2_, as a doubly occupied molecular orbital delocalized over the two atomic centers, reflects the independent motion of the two electrons. The reason this ansatz works well around *R*_e_ is that the relatively high electron density favors the lowering of kinetic energy by delocalization, but this is not so at larger *R*, where the lowering of potential energy by correlation becomes more important. Independent electron motion means large fluctuations in electron density including the presence of ionic configurations with both electrons at the same proton. This leads, in turn, to an erroneous asymptotic limit mixing covalent H–H with ionic H^+^–H^–^ and H^–^–H^+^ configurations in equal measure. This error is rectified by introduction of correlation, i.e., synchronized electron motion favoring the covalent configuration H–H of two neutral atoms.

Arguably, the simplest way of accounting for strong electron correlation is illustrated by the famous Heitler–London VB theory,^31^ which first provided a consistent quantum mechanical model of bonding in H_2_ and has seen much subsequent refinement.^32^ It is based on a simple correlated wave function, referred to as the VB, which omits these ionic configurations H^+^–H^–^ and H^–^–H^+^ from the Hartree–Fock wave function, retaining only the two covalent configurations, as if the electron pair showed a perfect left–right correlation. The VB model thereby produces a very good bond energy Δ*E*_b_(*R*) for H_2_ as shown in Figure 9. It may seem that the success of the VB method shows that electron correlation, rather than delocalization and interatomic electron motion, is the bonding mechanism of H_2_. We have noted that this is not the case^33^ and will confirm and clarify this observation below.

**Figure 8:**
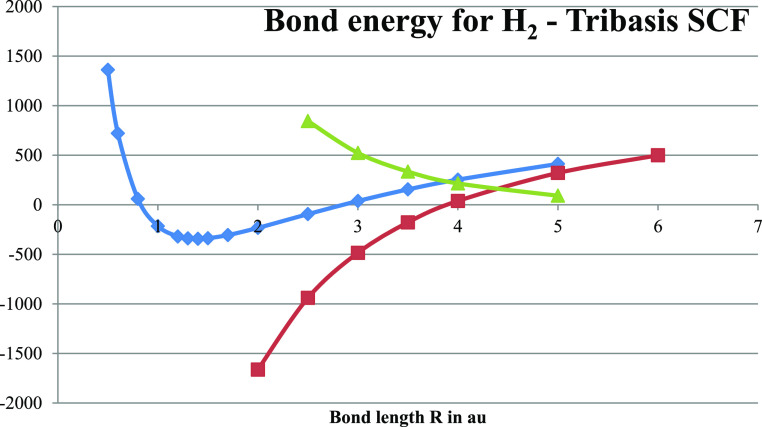
Local (triangles, one electron in each atomic subspace and no crossing) and delocalized (diamonds, electrons oscillating independently between atoms) bond energies of H_2_ (in kJ/mol) are shown as obtained from the tribasis Hartree–Fock analysis using the minimal (2 × 1s) basis set with the optimized (CI energy minimized) exponent. The delocalization energy (squares, difference between the two bond energies) is also shown.

**Figure 9:**
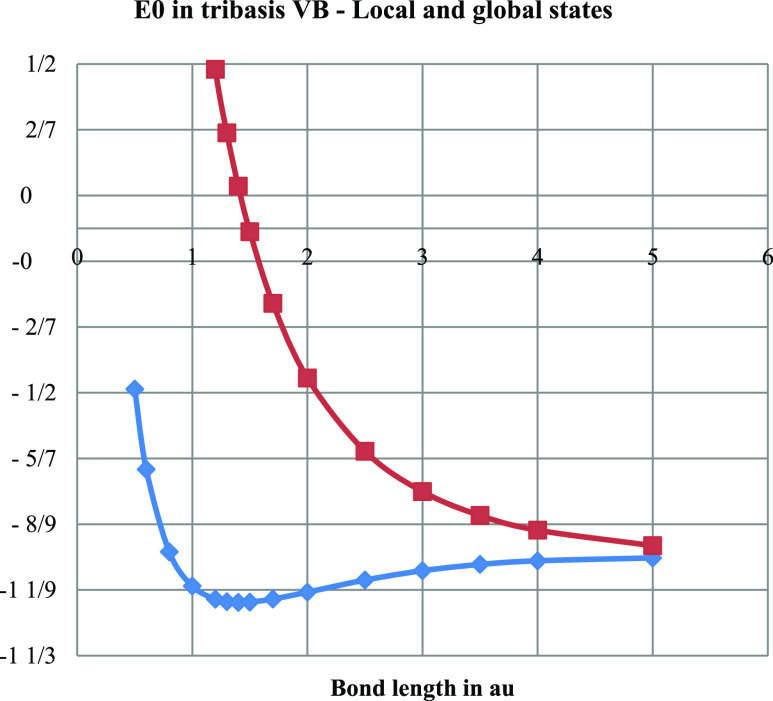
Energy (in au) of the local (squares, repulsive) and delocalized (diamonds, attractive) ground states in the tribasis implementation of the VB for H_2_ with the optimized (CI energy minimized) atomic basis exponent.

The picture of Hartree–Fock as a theory only applicable around *R*_e_, while perfectly left–right-correlated VB theory gives good bonding in H_2_ for all *R*, is simplistic and needs refinement. The success of the simple VB model is not only, not even primarily, due to the reduction of electron–electron repulsion that it brings about by comparison with the Hartree–Fock ground state. Instead, the overlap between the atomic basis functions used to produce the so-called covalent configurations plays the decisive role. This overlap allows kinetic coupling to be established so that the electrons are not strictly localized but show a synchronized motion between the two protons. Without this motion, the hydrogen atoms repel rather than attract. We showed this by using orthogonalized atomic orbitals in the construction of the covalent configurations.^34^ This eliminated the kinetic coupling between the two covalent configurations and thereby also the bonding. Thus, it is still the delocalization and interatomic electron motion which produce the bond, but it is combined with a correlation mechanism which is essential for the strength of the bond at larger bond lengths. Correlation is a restriction on delocalization, and it increases kinetic energy while reducing potential energy. There is a compensatory balance between delocalization and correlation which shifts from delocalization toward correlation as we move from *R*_e_ to larger bond lengths. Hartree–Fock theory in its usual form ignores this balance in always favoring full delocalization of independent electrons. For this reason, it can only provide a good representation of the bonding mechanism in H_2_ for relatively short bond lengths. The bonding in H_2_ is, more generally, due to a partially correlated delocalization. The delocalization, i.e., interatomic electron motion, remains the fundamental condition for bonding, but correlation is needed to describe its strength consistently, particularly for large *R* where it is weak.

The introduction of the tribasis reconstruction of the minimal basis set can add significantly to the picture mentioned above. First, we note that when Hartree–Fock theory in the full tribasis is used and only the delocalized eigenfunctions considered, then we reproduce the usual minimal basis results. The improvements due to “bridge optimization” are very small and insignificant in the discussion of the bonding mechanism. What is new and interesting is that we can now rigorously define a local initial state for a “dynamical delocalization” by omitting the bridge basis function. This produces local ground states with one electron on each side of the bond mid-plane and no passage allowed. We note that these local states, of high degeneracy due to free choice of spin direction, are common to both Hartree–Fock and the VB theories. They are like covalent configurations in the VB method but with orthogonality and without overlap. For this reason, they are perfectly localized and show no delocalization and no covalent bonding. Instead, they display the Pauli repulsion of localization, while the electron–electron repulsion, which is overestimated in delocalized Hartree–Fock theory, is reduced even slightly more than in the usual VB method. This follows since the localized states in Hartree–Fock theory have no orbital overlap and keep the electrons more separated than do the usual covalent VB configurations. For this reason, there is no ionic contribution, and for large *R*, the energy of the local ground state approaches the correct independent hydrogen atom limit. This means that within Hartree–Fock theory implemented in the tribasis, the local and delocalized state energies cross at a bond length of *R*_0_ ≈ 4 au beyond which the local state becomes the variational ground state. Thus, within Hartree–Fock theory, there is a localization of the bonding mechanism at *R*_0_ which allows the correct independent atom limit to be approached. This is a new feature not normally considered within Hartree–Fock theory; i.e., given the nature of the tribasis, the independent electron states of H_2_ generated include covalent VB configurations of dynamically localized character.

The VB method as applied to H_2_ is less influenced by the tribasis reconstruction. The localized VB initial states are the same as those in tribasis Hartree–Fock theory discussed above. It may seem that this local state is the covalent configuration in ordinary VB theory, but this is far from so. The ordinary VB method relies on the overlap between the atomic 1s orbitals to facilitate delocalization of the electron motion even in the correlated, seemingly localized, and covalent configurations. The tribasis has removed this overlap in the local ground state and with it also the stabilization associated with delocalization. The bond energy *E*_b_(*R*) is then repulsive due to localization of each electron to its own atomic half-space, i.e., due to the “Pauli repulsion of localization” discussed for H_2_^+^. The compensating reduction of electron–electron repulsion due to the left–right separation is a smaller effect. The full tribasis VB wave function, with three additional two-electron configurations (left-bridge, bridge-right, and bridge–bridge), produces the usual good bond energy curve of the minimal basis set calculation. The calculation was performed as a two-configuration configuration interaction (CI) with the ordinary VB and the local tribasis VB configurations as the basis set. Naturally, these configurations strongly overlap, but the resultant configuration, when the former is orthogonalized to the latter, gives a kind of “bridge configuration” with one or both electrons concentrated at the bond midpoint. A more complete calculation would include separate configurations for the cases when one or both electrons are in the bridge orbital, but given the success of the original VB method, we believe that the additional accuracy gained would be very small.

The dynamical delocalization energy, facilitated by the three VB configurations with one or both electrons in the bridge state, is composed of the bond energy minus the Pauli repulsion energy (in au)
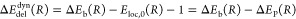
7

It is unlikely that the term “delocalization energy” has seen widespread use in the traditional treatment of H_2_ by VB theory given that the focus will have been on the reduction of electron–electron repulsion. The energy lowering would seem to come mainly from a correlation mechanism which reduces the electrostatic energy by a localization mechanism. We now know that this localization is incomplete due to the overlap of the atomic basis functions, and a correlated delocalization is present which is the cause of bonding.^34^ Eliminating the overlap, as in the local tribasis ground state, or orthogonalizing the atomic basis functions prevents interatomic electron motion and produces a repulsive bond energy. Again, we see (Figure 9 shown above) that the dynamical delocalization stabilization (the full spacing between the bond energies shown) is much stronger than the bonding mechanism itself in H_2_. The reason for this is that the Pauli repulsion is recognized and treated separately. This repulsion is due to the increasing cost in energy with smaller *R* of constraining the electrons to be either left or right of the dividing surface at the bond midpoint. The delocalization overcomes this repulsion and provides covalent bond energy by allowing the electrons to oscillate, in a correlated manner, between the protons.

The analysis of bonding in terms of stabilization by a mechanism of dynamical delocalization has clearly placed new focus on the Pauli repulsion mechanism which may seem to have an expanded meaning. Previously, Pauli repulsion may have been conceived as being related exclusively to effects introduced by the Pauli principle of wave functions fully asymmetric with respect to electron exchange. Here, we have noted that the operation of localization eliminates the need for a wave function in the form of a determinant since physical separation itself eliminates the exchange effects, and direct product wave functions will do. Thus, the Pauli repulsion we see here for left- or right-localized electrons in H_2_^+^ and H_2_ would arise also for a spinless electron. If we imagine a hydrogen atom in its ground state approaching a hard wall impenetrable to the electron, then the same localized ground state wave function would apply since it is determined by the boundary condition ψ(**r**) = 0 if **r** is in the surface of the wall. Apart from a minor difference due to imperfect screening of the “other” proton, the Pauli repulsion in H_2_ would be the same as that experienced by a hydrogen atom hitting a hard wall. Thus, we are using here a concept of “steric Pauli repulsion” which incorporates repulsion associated with electron exclusion from certain volumes. This type of repulsion, in the form of an “excluded volume effect”, is an important part of the bonding analysis and inescapable if we want to rigorously separate localized and delocalized states.

### 3.3 Some Reflections on the Nature of the Tribasis Analysis of Bonding

The analysis of bonding in H_2_^+^ and H_2_ carried out above is mainly of mechanistic character; i.e., it allows us to more precisely define what we mean by “delocalization” in a quantum mechanical calculation of the electronic ground state of a molecule and to relate this mechanistic concept to the dynamics of electrons. While wave functions, and in particular basis functions, are not observables in themselves, they can be chosen to yield physical insights into the mechanism of bonding. This is made possible by the basis set itself being “mechanistic”, in the case of the tribasis, by having a subset of local functions and a global function bridging the atomic subspaces. Thus, we have been able to clearly describe states of localized electrons and to turn on delocalization by adding the bridge basis function. For symmetric diatomic molecules, as studied above, the tribasis should, in principle, be based on the exact, or fully converged, orbitals of the molecule. We have been satisfied to explore the method in the minimal basis set which is known to provide a good ground state, particularly when the orbital exponent is optimized. We have noted that this “goodness of fit” is not equally applicable to the local ground state which is more closely related to the first excited orbital wave function of H_2_^+^ and H_2_. The exponent reflects the energy of the corresponding orbital, and at short *R*, these two orbitals are widely split in energy. The exponent optimized for the ground state is not optimal for the first excited state. Thus, we cannot expect excited state energies to be accurate when *R* is around the equilibrium value and below where the orbital contraction is significant. For such low *R*-values, we should treat the excited state energies, and the related delocalization energies, as qualitative. This limitation is, of course, relevant to our minimal basis exploration but not to the tribasis analysis itself.

**Figure 10:**
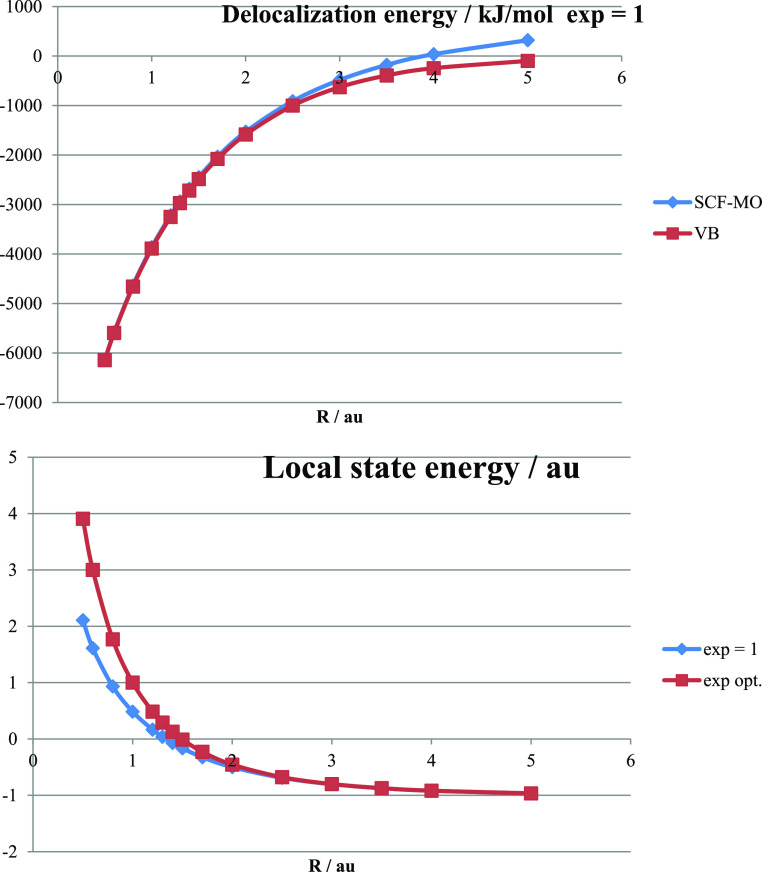
Delocalization energy (top pane) in kJ/mol obtained with the unscaled H 1s basis (ζ = 1) for H_2_ in Hartree–Fock and VB implementations of the minimal tribasis. The effect of ζ-scaling for ground state optimization (CI energy) on the local ground state (the same in SCF-MO and VB) energy (in au, the energy unit is 1 hartree = 2625.5 kJ/mol) is also shown (bottom pane).

In order to get some understanding of the errors arising (see also Figure 5 for H_2_^+^) when we optimize the exponent ζ to suit the delocalized ground state (here done with respect to the CI energy) and then use it for the calculation of the local ground state as well, we include the delocalization energies in the Hartree–Fock and VB calculations with ζ = 1 as shown in Figure 10. We note that these two delocalization energies agree closely for small bond lengths *R*, where the scaling is most significant, but a comparison with the results for optimized ζ as in Figures 8 and 9 presented above shows that the magnitudes are smaller for ζ = 1. Obviously, the local state is not well served by the “orbital contraction” of the delocalized ground state. This phenomenon of scaling exaggerated error of the local state is, however, confined to *R* ≈ *R*_e_ or less. It does not affect our qualitative analysis of the origin of bonding in delocalization. The most interesting feature seen in Figure 10 is the divergent behavior of delocalization for larger *R*, i.e., the clear evidence that independent electron (Hartree–Fock) delocalization becomes antibonding around *R* ≈ 4 while the correlated delocalization of the VB method remains bonding for large *R*. The effect of the scaling of ζ to optimize the delocalized ground state energy on the local ground state energy is also shown. The dramatic increase in local state energy for small *R* demonstrates that the orbital contraction needed to improve the ground state is entirely dependent on the delocalization. Local ground state orbitals do not contract in the same way as the molecule forms do.

**Figure 11:**
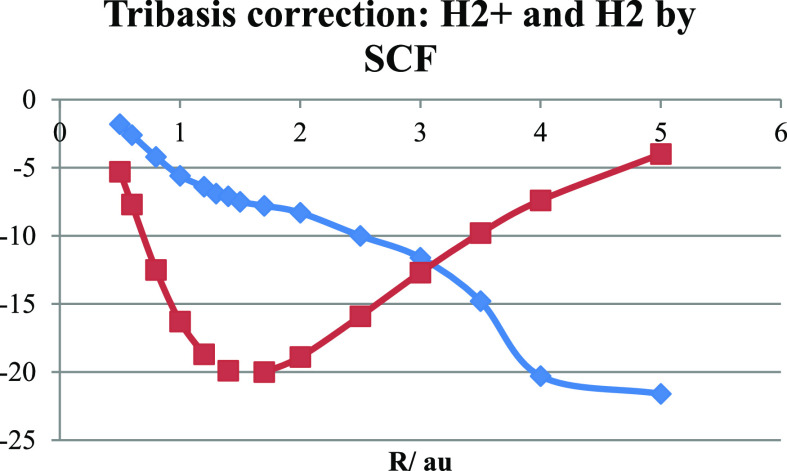
Tribasis corrections to bond energies (in kJ/mol) of H_2_^+^ (squares) and H_2_ (diamonds) within Hartree–Fock theory both implemented in the minimal basis with optimized exponent ζ.

A related issue of interest in the tribasis analysis is the accuracy of the ground state and of the corresponding bond energy. If we were able to determine the exact ground and first excited state orbitals of H_2_^+^, then the ground state and the first excited states would be exactly reproduced by construction. Thus, the issue of accuracy relates for H_2_^+^ to approximate implementation like that in the minimal basis employed here. In the case of H_2_ and larger molecules, the model used to describe electron correlation will also play a role. We can, as noted already, immediately deduce that the tribasis results will be better than those in the original minimal basis. This follows by the variational principle and the fact that the function space spanned by the tribasis includes the space of the minimal basis. How much better are the ground state results for H_2_ in our minimal basis treatment? The corrections are shown in Figure 11. The answer is “not much better”. On the scale of the bond energy itself, the further stabilization achieved by the tribasis is no more than about 20 kJ/mol which is barely significant. The maximal bond strength for H_2_^+^ goes from 227 to 246 kJ/mol at *R* = 2.0 au, while for H_2_ by the Hartree–Fock method, the same maximal strength goes from 336 to 343 kJ/mol at *R* = 1.4 au by the tribasis correction. In the case of H_2_ treated by the VB method, the tribasis corrections are much smaller and may not be numerically significant. The VB bond strength is found to be 363 kJ/mol at *R*_e_ = 1.4 au with a tribasis correction of only 1 kJ/mol.

The results clearly show that the tribasis correction due to the ability, afforded by the bridge basis function, to optimize the wave function around the bond midpoint is most important in the case of H_2_^+^ where it follows the bond energy curve with a slight shift toward shorter bond length. The behavior is entirely different for the Hartree–Fock calculation of the H_2_ bond energy where the correction increases in magnitude with *R* reaching 20 kJ/mol by 5 au. We note that this means 10 kJ/mol per electron, which is smaller than the maximal correction for H_2_^+^. We believe that the explanation for the difference can be found in the shape of the potential seen by the electrons. In H_2_^+^, the potential is that of two protons and therefore twice as negative at the bond midpoint compared to the corresponding value at the same distance from a single proton, as in the atom. Thus, the basis function, being adapted to the single proton, is too small to resolve the accumulation at the bond midpoint. This is partially compensated by the optimized coefficient of the bridge function in the molecular orbital. In the case of H_2_, there is screening by the “other” electron which reduces the effective one-electron potential at the bond midpoint. In the case of the VB treatment, this screening is nearly perfect so that there is no significant “nonatomic behavior” at the bond midpoint and little need for tribasis correction. Finally, it should be noted that if the minimal basis treatment is considered good enough, the tribasis can be used without bridge function optimization, i.e., accepting the original minimal basis form of the delocalized ground state. This elimination of an optimization step could speed up calculations at a relatively small cost in loss of accuracy.

## 4 Tribasis Extension of Hückel Theory

The tribasis analysis of our smallest molecules H_2_^+^ and H_2_ mentioned above can, and should, be followed by extensions to larger homogeneous diatomic molecules and, after generalization to account for the asymmetry and admixture of ionic bonding, to molecules of any structure. Here, we shall start out on this path toward generalization by revisiting^24^ Hückel theory of π-electron bonding in aromatic molecules. This theory is of special interest since it illustrates the role of orbital delocalization for both electron motion and covalent bonding in what seems to be the simplest possible way. Previously, we have attempted to bring Hückel theory closer to ab initio quantum chemistry by accounting for the overlap between the atomic orbitals used in the basis set. Now, we shall apply the tribasis method presented above first to determine the dynamical delocalization energy and the Pauli repulsion of localization from overlap-corrected Hückel theory and second to extend the theory to the tribasis form where not only atomic sites but also bridges between sites are represented by specific basis functions. This allows both greater accuracy and a deeper probe of the mechanism of π-electron bonding.

### 4.1 Hückel Analysis of π-Bonding in Ethylene

One can say that Hückel theory is based on a simplified one-electron model of the π-bond in ethylene which is then extended to a theory of π-electron orbitals, energies, and bonding in larger networks of π-bonds by the assumption of uniform local site and site–site coupling parameters. A model diatomic π-bond in this way generates a “lattice theory” of π-electron bonding in extended molecular structures. We shall keep this general approach but improve the underlying diatomic model to account for overlap and dynamical delocalization.

#### 4.1.1 Traditional Hückel Analysis

The traditional Hückel representation of the π-bond in ethylene CH_2_=CH_2_ sets it out as a coupled two-state problem in the basis of the two C 2p_*z*_ atomic orbitals with the Hamiltonian and energy eigenstates and eigenvalues
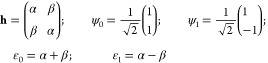
8

We recall that α is the interaction energy of the π-electron localized on a single atom with the rest of the molecule, and β is the negative coupling parameter which allows it to move between the atomic centers. Since it is a one-electron model, the bond energy (for the electron-pair bond) is
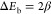
9which is also the total delocalization energy in the traditional parlance. In principle, the Hückel model can be applied for any bond length *R* between the carbon atoms, but in practice, it is nearly always used for an assumed equal bond length of a C=C bond or corresponding aromatic bond. The coupling parameter β will naturally go to 0 as *R* → ∞, but in a simplest interpretation of the model, α is independent of *R* and equal to the C 2p-orbital energy of the atom.

An explanation is needed here given that no mention is made of the nuclear repulsion nor of any electrostatic interactions that play such an important role in other forms of quantum chemistry. This absence of electrostatics is a feature of the Hückel models which implicitly rely on a “perfect screening assumption” that cancels such effects and leaves the bonding to be due to electron delocalization alone. This is of course not quite correct, but given that variations of bond length *R* are normally not considered, the errors can, to a degree at least, be absorbed in the model parameters. This total focus on electron delocalization to the total exclusion of electrostatics will be true for all Hückel models discussed here. It is a great simplification but also a reason why Hückel theory is difficult to derive directly from ab initio quantum chemical theory.

#### 4.1.2 Overlap-Corrected Hückel MO (ocHMO) Method

The above-mentioned problem of the neglected overlap can be corrected rather easily^24,35^ by simply recognizing mathematically that the two (left and right) atomic orbital basis functions φ_l_(**r**), φ_r_(**r**) have an overlap integral equal to *S*, i.e.,
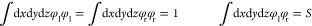
10

Upon inserting this into the Schrödinger equation, we find
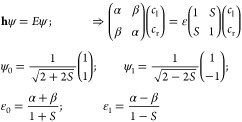
11

Note that the complication due to overlap is very small since it does not alter the form of the eigenvectors, only the eigenvalues. Given that *S* is positive and in the range 0 < *S* < 1 and α is negative, we see that the bond energy is reduced by the overlap of the atomic orbitals. This was confirmed in calculations performed in our earlier work.^24^

It is interesting to note that the π → π* transition energy in the original and overlap-corrected models is
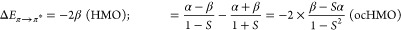
12

The corresponding expressions for the bond energy are
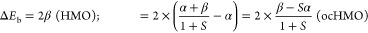
13

Thus, we see that the ratio between bond energy and π → π* transition energy goes like
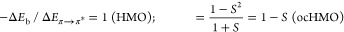
14

Clearly, this flexibility obtained by introduction of a parameter accounting for orbital overlap represents a considerable improvement on the original Hückel model. We see that an overlap coefficient S can be adjusted to account for a “caloric–spectroscopic divergence” of bond (caloric) and transition (spectroscopic) energies from the zero sum (i.e., equal in magnitude but opposite in the sign) predicted by the original Hückel model.

#### 4.1.3 Recovery of Dynamical Delocalization by Tribasis Relation

Recall that for H_2_^+^, the tribasis analysis identified the local ground state energy as the same as that of the antibonding first excited state and the dynamical delocalization energy as the full energy difference between the antibonding and delocalized bonding state. This result will hold for all symmetric one-electron systems. In particular, it will hold for the π-electrons of ethylene. Thus, we can take over the systematic results of the analysis of H_2_^+^ to the Hückel model of ethylene. This will be done below.

It has been noted above in the overlap-corrected Hückel analysis that orbital overlap introduces a “Pauli repulsion” between atoms which reduces bond energy. An account for this overlap, as outlined just above, brings the Hückel analysis in contact with quantum chemistry both theoretically and with respect to results obtained.^24^ One can see that π-bonding in ethylene is mechanistically related to a delocalization (or interatomic electron motion) mechanism overcoming a repulsion related to orbital overlap (or constriction of available space associated with orbital overlap). We now apply the tribasis scheme, described above for a pair of hydrogen 1s orbitals in H_2_^+^, to the two carbon 2p_*z*_ orbitals of the Hückel analysis of ethylene. The antibonding combination of these two orbitals will, due to symmetry along the bond axis, again have a node at the bond mid-plane allowing the left and right lobes to define local basis functions as mentioned above. It immediately follows that the tribasis analysis leads to a localized state energy equal to that of the antibonding state of the overlap-corrected Hückel model outlined above.

Given that the function space spanned by the “π-tribasis”, consisting of the two antibonding lobes mentioned above and the bonding ground state, contains the full function space of the two 2p_*z*_ orbitals, the ground state of the overlap-corrected Hückel (ocHMO) model is contained in that space, and we can accept that as our ground state in our bonding analysis, i.e., ignoring the (presumably) small stabilization associated with bridge function optimization. If so, we have identified a two-step mechanism of bonding as follows:

Step 1. Starting with two noninteracting π-electrons (*S* = 0, β = 0), we let the atoms approach, but the electron remains localized to its own atomic half-space (*S* > 0, β < 0). The π-electron energy then goes from α to (α – β)/(1 – *S*)
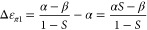
15

Step 2. With the π-electron localized, the bridge function is added and the delocalization turned on to bring the electron to the ocHMO ground state of energy (α + β)/(1 + *S*).
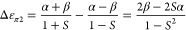
16

Note that these two steps are just the one-electron Pauli repulsion and delocalization energies, respectively. The total π-bond energy is then obtained as a sum of these two step contributions for the two electrons in the bond.
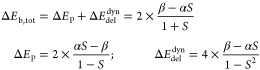
17

Finally, we note that the Pauli repulsion and delocalization energies are related to the bond energy contribution by a scaling dependent only on the overlap factor *S*, i.e.,
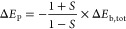
18

We have now resolved the π-bond energy of ethylene according to overlap-corrected Hückel theory into a bonding contribution due to dynamical delocalization (electron motion between atomic subspaces) and an antibonding contribution due to a type of Pauli repulsion associated with the localization constraint and the elimination of overlap between atomic orbital basis functions. We note that if we set *S* to 0 in these expressions, we return to the original Hückel model HMO. Unfortunately, this limiting case is unphysical except in the limit of weak coupling, e.g., by *R* → ∞. The ratio of Pauli repulsion and delocalization energy can be obtained as follows
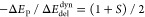
19

Thus, we see that as suggested above, the Pauli repulsion amounts in magnitude to about half of the delocalization stabilization for small overlap *S*, but as *S* grows for shorter *R*, it approaches the same magnitude.

**Figure 12:**
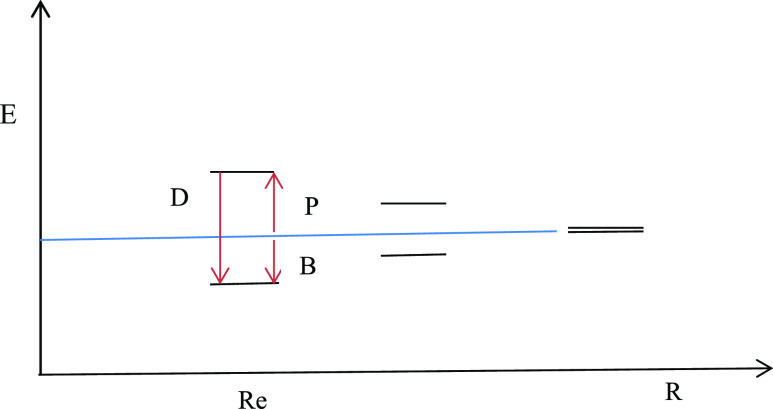
The dynamical mechanism of covalent bonding is shown for one-electron models of covalent bonding. The splitting of ground and first excited state orbital energies is shown for three different bond lengths. The excited state (antibonding) is also the localized ground state. The dynamical delocalization energy (D in graph) is identified by the long arrow from the excited to the ground state. The bond energy (B) is shown by the short arrow to the right from separate atom to ground state molecular energy. The arrow above it, from separate atom to first excited state energy, represents the Pauli repulsion energy of localization (P).

A relatively simple picture emerges which will have validity far beyond the examples so far studied: In an orbital-based description of the covalent bonding of a homogeneous diatomic molecule, a rise in energy equal to that of the antibonding orbital ε_1_ is associated with the localization of electron motion to an atomic half-space, while the drop in energy from antibonding to the bonding orbital is associated with dynamical delocalization, i.e., interatomic electron motion. Figure 12 illustrates the one-electron mechanism of bonding arising from the proposed analysis in terms of the electron dynamics being localized to individual atoms or delocalized over the two atomic centers.

This picture is rigorously correct for one-electron systems like H_2_^+^ and one-electron models like the overlap-corrected Hückel model of ethylene. It will most likely have bearing also for many-electron models like the Hartree–Fock or Kohn–Sham DFT approximations, but as we saw for H_2_ above, the electron correlation mechanism is expected to favor the localized electron configurations and thereby reduce the stabilization achieved by delocalization. Thus, the correlation mechanism will require an addendum to the picture of covalent bonding as a sum of two mechanisms: a repulsion due to electron localization to atomic half-spaces followed by a stronger attraction due to relaxation of this constraint and electron motion between atomic subspaces. The electron correlation implies a localization, but it is only partial, and as indicated by our study of H_2_ by a VB method mentioned above, the interatomic motion of electrons remains the key mechanism producing the bonding.

### 4.2 New Hückel Model in the Tribasis Form

We have seen above that traditional Hückel theory neglects the nonlocality of the atomic orbitals in its basis set. By overlap correction, the corresponding error is avoided, and by the tribasis analysis, we have shown that for ethylene, the bond energy can be resolved into a localization repulsion and a dominant delocalization attraction. The success of this analysis suggests that the Hückel model be reformulated in the tribasis formed from the original Hückel basis of C 2p_*z*_ atomic orbitals. This would have the advantage that the basis set can be assumed orthogonal with only the small error due to overlap with next nearest and further removed atoms. The structure of model theory will thereby be very realistic, and the energy eigenfunctions will reveal whether the bridge basis functions serve (1) as virtual agents of infrequent interatomic barrier crossing or (2) as dominant shape changers of the molecular orbitals to “bond central” form. Certainly, the former must be the case for large atomic separations, but how much of the latter is present at molecular equilibrium geometry?

#### 4.2.1 Diatomic tHMO Model

The tribasis reformulation of Hückel theory for ethylene uses a basis set consisting of left- and right-localized functions φ_l_, φ_r_ and a bridge function φ_b_ which are orthogonal and can be taken to be normalized to unity. They are formed from the underlying minimal basis set for π-bonding of left and right C 2p_*z*_ atomic orbitals by the exact same steps as those applied to the H 1s orbitals in the example of H_2_^+^ mentioned above. The ethylene π-electron Hamiltonian in the new basis is
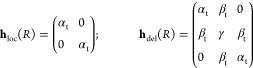
20

Here, the first form is applicable if the electron is localized on either side of the bond mid-plane, and the second form arises by addition of the bridge function which couples the two atomic subspaces and allows interatomic motion. The subscript t has been added to α and β to remind us that these parameters do have other meaning and values in tHMO than those in HMO theory or its overlap-corrected version ocHMO. The parameter α_t_ is the energy of the electron when localized to the left or right half-space and β_t_ is the coupling matrix element between such a left- or right-localized state and the bridge function. The parameter γ is the energy of the electron in the bridge state. The two Hamiltonians correspond to the localized and delocalized electron motions in the diatomic molecule. Taking the lobes of the antibonding combination of C 2p_*z*_ atomic orbitals to be sign-shifted to be mirror-symmetric in the bond mid-plane, we see that while **h**_loc_ has two localized energy eigenfunctions φ_l_ and φ_r_ of degenerate (equal) energy, in the delocalized case, their antibonding combination  will be an energy eigenfunction, but it will be nondegenerate since there are two other delocalized eigenfunctions, i.e., the ground state and a third symmetric state of double node character. Thus, we have a trivial antisymmetric state of energy α_t_ and two symmetric states of as yet unknown energy. The ground state will have a form given by symmetry, so we can easily work out the ground and first excited state orbitals and energies
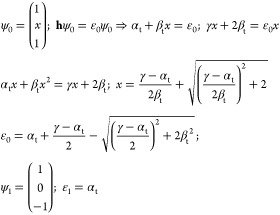
21

We note that the delocalization energy for a pair of π-electrons is
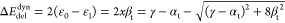
22

In order to obtain the localization energy and the total bond energy for the π-bond, we need to know the uncoupled (*S* = 0 and β_t_ = 0) single-electron energy α_t_(∞) which should be equal to α in the HMO and ocHMO theories. We get
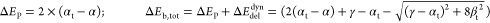
23

This result is based on properly localized and orthogonal basis functions derivable from quantum chemistry at any bond length. It establishes the mechanism of covalent bonding as a result of stronger delocalization attraction overcoming a Pauli localization repulsion as the atoms approach.

#### 4.2.2 Extension of the tHMO to Larger Molecules

Original Hückel theory is clearly a model of an empirical nature. The basic model is taken from quantum mechanics of noninteracting electrons, but the assumptions of the model, the neglect of overlap in particular, are not so much derived as postulated, and the parameters are generally fitted to experimental properties. The kernel of the theory is the treatment of a two-electron two-site π-bond C−π–C. Then, the model is empirically extended to bonding in structures of three or more sites, with one electron per site, by the assumption that the site energy α and coupling between neighboring sites, i.e., β between nearest neighbor sites and no coupling between non-nearest neighbor sites, remain the same. Thus, end sites and inner sites are taken as fully equivalent as well as the coupling between them. This means that the Hückel Hamiltonian is very easily constructed for larger structures that we shall now consider. The tribasis extension proposed above is based on the assumed existence of a one-electron potential providing a good representation of bonding in the two-site kernel. For this kernel, the model is derived with full account of the overlap between atomic basis functions. The kernel is then used to generate the extensions to larger chains and rings by the same assumption of site and bridge equivalence as that used in original Hückel theory. The resulting tHMO model is therefore much like the original model in its construction but fundamentally different in the explicit treatment of atomic basis overlap in the two-site kernel and can therefore claim to describe dynamical delocalization. Errors remain mainly in the empirical treatment of electrostatics and electron correlation but also in the lack of distinction between inner and external sites in chains.

##### 4.2.2.1 tHMO for Molecular Chains and Rings

We now extend the analytical exploration of our tHMO model from the C−π–C bond to larger molecular structures. This will be done, as in the original HMO model, on the assumption that the site, bridge, and coupling parameters can be taken to be the same in a chain or ring as those in the diatomic reference molecule. This is, as noted, not quite correct, but it is practical since it gives the model the character of lattice theory of minimal parameterization. A more accurate model would distinguish sites and coupling parameters depending on whether they were relating to within chain or end sites, which differ most clearly by the degree of localization constraint, but we leave such considerations for future study. Instead, we employ the uniform site assumption, explore the predictions for larger chain and ring structures by analytical mathematics, and allow the empirical fits to make the best of the parameterization in this ensemble of structures.

##### 4.2.2.2 Three and Four Atomic Structures

We consider now planar hydrocarbon structures of three and four carbon sites which may be chains or closed regular triangles or squares, i.e., isomers of allyl radical or butadiene. We do not, for the present purpose, account for the stability of the various corresponding σ-structures but focus our attention on the π-electron contribution to the stability. In order to remind us of this simplified Hückel modeling of molecular π-electron structures, we refer to the carbon atom sites as Hü atoms. Later on, we match them up with real molecules as needed.

We begin our exploration of larger molecules with linear (l) and cyclic (c) forms of Hü_3_. This 3-electron problem will include exchange correlation between the two electrons of the same spin. Within our one-electron model, this will have no effect beyond the Pauli principle exclusion of more than two electrons in the same orbital. Similarly, the parameters α_t_, β_t_, and γ should be revaluated for bond–bond interactions at angles other than 180°. Changes are expected due to intersecting bond mid-planes, but in keeping with the original Hückel model, we ignore this also for the moment in order to explore our simplified lattice model. The linear (l) and cyclic (c) Hamiltonians are then as follows
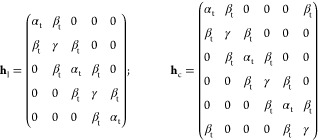
24

The lowest eigenvectors, ground and first excited, can be found analytically, and we get the following energy eigenvalues
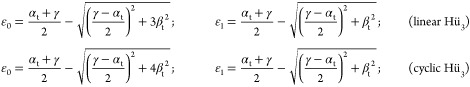
25

With two electrons in the ground and one in the first excited orbital, the total bond energy is
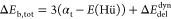
26

Here, *E*(Hü) is the separate atom energy α as mentioned above, and we note that the first term on the right-hand side is the Pauli repulsion of localization. The dynamical delocalization energies can then be found as follows
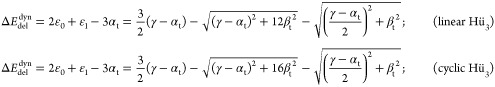
27

We note that the total bond energy is composed of a delocalization attraction and a Pauli repulsion. The latter is due to the cost in energy of localizing the electron motion which was ignored in traditional Hückel theory. For linear Hü_3_ in particular, this term may possibly be large enough to make the molecule with equal bond lengths less stable than the equal length chain of a Hü_2_ molecule with a separate “Hü atom”. In the case of the cyclic molecule, the delocalization energy is stronger, but the radical character may still lead to weak bonding in the fully symmetric molecule.

Normally, the geometry of the “Hückel molecule” is determined by the network of underlying σ-bonds, but it is possible to apply the Hückel analysis at any geometry. In the limit of large separation between atoms *R*, the coupling parameter β_t_ vanishes, but α_t_ does not, and we can expand the square roots according to the relation
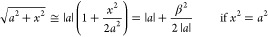
28

Employing this relation, we can simplify the expressions for the delocalization stabilization in the large *R* limit as follows
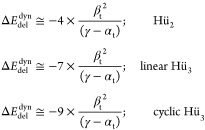
29

We note that for linear Hü_3,_ the delocalization energy per electron is asymptotically just marginally greater than that for Hü_2_; i.e., it is larger by only the factor 7/6 due to the increased chain length. The closing of the chain into a ring in cyclic Hü_3_ does increase the value by a factor of 3/2, reflecting the larger number of bonds in the structure.

A more suitable molecule to study, given its complete symmetry and even number of electrons, is cyclic Hü_4_ where the Hückel atoms form a square, and each site contributes just one 2p_*z*_ electron. Again, we recall that localization will be influenced by the angular deviation from the straight chain, but we ignore this and stick for the moment with our simplest lattice model of the electronic structure. We note also that the four electrons can form two pairs. There is no radical character, but full electron pairing is available for the four electrons which is what we expect in a stable molecule. The Hamiltonian and the ground and first excited π-orbitals for cyclic Hü_4_ are as follows in our tribasis
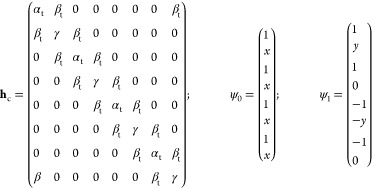
30

The eigenvalue equations are readily solved for cyclic Hü_4_ to yield
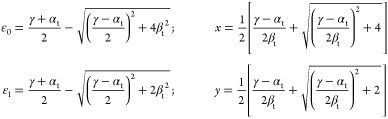
31

The total π-electron energy for cyclic Hü_4_ is
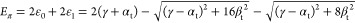
32

The localized ground state energy is just 4α_t_, so we can readily obtain the delocalization energy
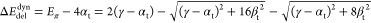
33

In the limit of large bond length *R* and weak coupling β, this relation simplifies to
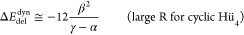
34

The “aromaticity”, as it is called in connection with π-bonding in organic chemistry, i.e., the energy of further delocalization beyond local pair bonds, is readily obtained by subtracting the energy of two independent Hü–Hü bonds from the total bond energy
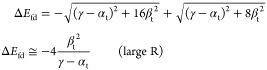
35

We see that there is an added stabilization due the “beyond pair-wise delocalization” in cyclic Hü_4_.

#### 4.2.3 From “Hückel Molecules” to Planar Hydrocarbon Molecules

The Hückel molecules mentioned above have counterparts among planar alkene molecules with a contribution from π-bonding which is separable from that from the σ-bond structure, as exploited in original Hückel theory. The main difference between the Hückel model and hydrocarbon reality is that the σ-bond structure plays a dominant role in the latter. It is stronger by comparison and imposes longer bond lengths than would be optimal for the π-bonds. This will reduce the interactions of the π-electrons. The ethylene molecule C_2_H_4_ is the reference molecule for the ensemble of planar alkenes whose π-bonds are studied by HMO theory. The results in eq 21 for Hü_2_ carry over to the π-bond in ethylene. We just note that the parameter value α_t_ is determined by the occupied σ-orbitals and the parameters β_t_ and γ by the equilibrium bond length *R*_e_ arising for ethylene due mainly to the C–C σ-bond. Thus, the parameters lock the model into an underlying reality. This reality will vary, but we shall assume, for the present, that the bond length and parameter values, α_t_, β_t_, and γ, remain fixed and the same for all the π-bonded alkenes we study.

The building block for our planar alkenes is the sp^2^p_*z*_-structured methyl group CH_3_ which bonds by replacing a hydrogen by a σ-bond and then forms a π-bond. In ethylene, we then have H_2_C−σπ–CH_2_. The addition of another methyl group leads to the allyl radical CH_2_CHCH_2_ which corresponds to Hü_3_. We note that our modeling implies that the replacement of a C–H with a C–C σ-bond does not alter the carbon site significantly. A fourth methyl group yields butadiene in a linear or cyclic form. The molecule cyclobutadiene (−CH−)_4_ corresponds to the cyclic Hü_4_ molecule, so we can take over the analytical results from above. We note that significant aromaticity, in the sense of stabilization due to delocalization over four rather than two separate pairs of carbon atoms, is predicted.

The cyclobutadiene molecule has all the symmetries which are needed to make the treatment of π-electrons straightforward, but the σ-bond network is still strained since the angles are forced to be 135, 90, and 135° around each carbon atom rather than the preferred 120, 120, and 120° according to simple bonding theory. The benzene molecule C_6_H_6_ can eliminate this σ-electron strain since the bond angles are optimal for the sp^2^p_*z*_-hybridized CH radicals. The cyclic structure of C_6_H_6_ leads to a ground state orbital with a uniform expansion coefficient over all sites, and the doubly degenerate first excited wave function will have the form of two independent linear allyl structures single-bonded to each other at each end. Thus, we can deduce that the orbital energies will be as follows
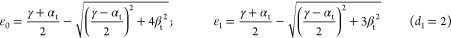
36

Note that the ground state orbital is nondegenerate (*d*_0_ = 1) but the first excited orbital doubly degenerate (*d*_1_ = 2). The total π-electron energy and delocalization energy are then obtained as
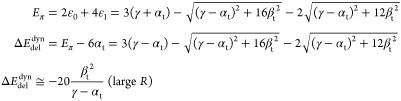
37

Finally, we find the aromaticity, defined as the energy of further delocalization beyond diatomic π-bonds, as follows
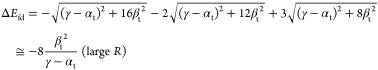
38

In all our discussions mentioned above, we have assumed that the C–C bond lengths are all the same and equal to those in ethylene from which we obtain our parameters α_t_, β_t_, and γ. We have noted that the bonds are strained in the case of cyclobutadiene. In fact, by experiment, the bond lengths are found not to be the same in the ground state of cyclobutadiene where two opposite bond lengths are found to be shorter and connected with two slightly longer bonds in a rectangle rather than a square of carbon atoms.^36^ This is generally understood as a result of the “4*n* + 2 electron rule” of aromaticity and Hückel theory, where *n* is an integer. We note that cyclobutadiene does not obey this rule since it has four π-electrons, but it is also exposed to Hund’s rule effects (triplet state formation) and Jahn–Teller distortion that go well beyond Hückel theory.^37^

A more systematic examination of the experimental deviations from the simplest form of tribasis Hückel theory that we have applied here will have to wait, but we would like to point out an important difference of the cyclobutadiene molecule when compared with benzene which obeys the 4*n* + 2 rule with *n* equal to unity. We see that for both molecules, the ground states are nondegenerate, but the first excited states are doubly degenerate. In cyclobutadiene, we doubly occupy just one of the two excited orbitals. These orbitals are “bond-inequivalent”; i.e., they localize π-bonds and thereby become susceptible to forces seeking to tighten them at the expense of increased single bond lengths. Experimentally, this is what is observed. In benzene, this does not happen because there are four electrons to place in two degenerate excited π-orbitals which together are uniformly distributed around the ring. Thus, there is no driving force toward bond inequivalence in benzene. It is interesting to note that our tHMO model, given its closer connection to a priori quantum chemistry, can, in principle, describe *R*-dependence and could therefore be used to resolve such shifts of bond lengths as seen in cyclobutadiene, if the corresponding *R*-dependence of σ-bond energies and site energy α_t_(*R*) were also available.

### 4.3 Empirical Parameterization of the tHMO Model for Hydrocarbon π-Bonding

The analytical results obtained above within the tribasis extension of Hückel theory of π-electron delocalization depend on three parameters α_t_, β_t_, and γ. It is possible, in principle, on the strength of a one-electron potential which can account for the π-bonding in ethylene, to directly calculate these parameters. This could be seen as an extension of the relationship between the overlap-corrected and tribasis forms of Hückel theory noted above. Here, we shall instead suggest how the needed parameters could be empirically determined. This approach is familiar from the use of the original HMO model, but one recalls also the difficulty that “caloric and spectroscopic” data fitting gives very different Hückel parameters α and β.^34^ Here, we need to fit three parameters in order to implement the tHMO model which means that we need more data. We shall assume that the explicit treatment of overlap allows us to use both spectroscopic and bonding data to fit our parameters.

#### 4.3.1 Empirical Data for Carbon π-Electrons

There are two types of data that we are primarily interested in: (i) bond energies and (ii) energy differences between π-orbitals. The former are the ultimate aim of bonding theory, and the latter have direct mechanistic significance in all the Hückel models discussed. Both these types of energies are directly measurable, although minor corrections may, as we shall see, be needed in order to make the most readily available data precisely of the form needed for our Hückel models. Here, we are concerned mainly with basic principles and will be satisfied with semi-quantitative accuracy, so minor corrections will not be pursued. There is, however, a rather significant difference between the two types of data which we must take note of, i.e., the different dependence on the overlap correction that they display. As we shall see below, this correction reduces bond energies very significantly but leaves the splitting between π-orbitals relatively less affected.

The data that we shall chose to use to determine the parameters of the tHMO model, α_t_, β_t_, and γ, will be the π → π* transition energy for ethylene, the bond energy contribution from the single π-bond in hydrocarbon molecules, and the aromatic stabilization of benzene beyond that afforded by the Kekulé structure. The energy gap between the ground and first excited π-orbitals in ethylene  can be observed by spectroscopy, but the excited state may then include a small shift in bond length. The vertical (fixed bond length) transition that we need here is readily studied by quantum chemical calculations.^38,39^ Theoretical results for the vertical transition energy center on 8.0 eV or 0.294 au or 772 kJ/mol (G. Bacskay, private communication). This value is close enough to the experimental transition energy, which may not be vertical, for us to feel justified in using it as our empirical vertical π → π* excitation energy for ethylene.

Given the exploratory nature of this work, we shall defer a more detailed study of bond energies and be satisfied with estimates obtained from a table of average bond energies^40^ (traceable back to our first reference: The Nature of the Chemical Bond by Linus Pauling^1^). The average carbon–carbon bond energies are listed in Table 1 as follows:

From the first two entries, we can see that a single isolated π-bond on average is found to contribute the binding energy
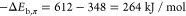
39

Turning to the aromatic stabilization of benzene, we see that according to the Kekulé structure of benzene with three local π-bonds, we have a total bond strength of
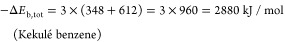
40while for aromatic benzene, we get
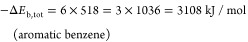
41

It follows that we get a total aromatic stabilization of benzene by
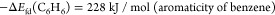
42

It is clear that this estimate is simplistic and could be improved by more refined data specifically for benzene, but we accept it for the present.

#### 4.3.2 Fitting of Hückel Parameters—The Spectroscopic–Caloric Conundrum

The traditional Hückel model has been found to yield a π → π* energy −2β and a single π-bond energy 2β. The ground state of benzene is easily seen to have a lowest orbital energy ε_0_ = α + 2β, and the first excited state is doubly degenerate with energy eigenvectors (not normalized) and eigenvalues as follows
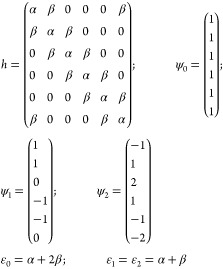
43

We see that the six π-electrons of benzene will doubly occupy all these three orbitals and yield a bonding energy contribution
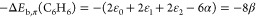
44

The Kekulé structure will yield
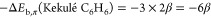
45

It follows that the further delocalization of the π-electrons in the ring of carbon atoms produces an aromatic stabilization
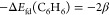
46

The fitting of Hückel’s β from our data then looks as follows
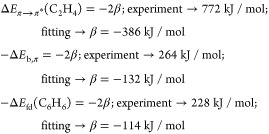
47

We see that the two bond energies give much lower β-values of −132 and −114 kJ/mol, while the spectroscopic π → π* transition energy is approximately 3 times larger in magnitude. Clearly, the atomic orbital overlap, which is missing in the original Hückel model, makes the simultaneous fitting of caloric (bond) and spectroscopic (transition) energies fail dramatically. This is seen in the results obtained by overlap-corrected Hückel theory which were compared with ab initio quantum chemical results in earlier work.^24^ This disparity poses a conundrum for the original HMO model which produces very useful results related to the nature of π-electron orbitals and their delocalized motion but still fails badly with respect to the quantitative relationship between bonding and transition energies. The use of a less simplistic HMO model with bond length-dependent α may mitigate this failure but not entirely remove it. A better remedy would be to use the overlap-corrected ocHMO model which will certainly improve the consistency of the parameter fit.

#### 4.3.3 Determination of Tribasis Hückel Parameters

The new tHMO form of Hückel theory yields the following relationships for our three chosen empirical π-electron energies
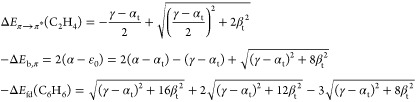
48

We note that the first and last equations, relating to transition energy and aromatic stabilization, depend only on two quantities
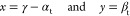
49

The middle equation adds a dependence on α_t_ – α which is the Pauli repulsion suffered by a single localized π-electron as the bond length is reduced from infinity to the value in the equilibrium π-bond. Thus, the fitting will proceed accordingly starting with the first and the last equations. They can be written in kJ/mol as
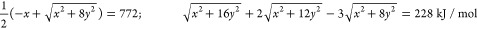
50

Defining  and assuming that γ < α_t_ so that x is negative, we can simplify these equations to the form
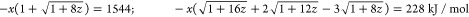
51

We can now take a ratio of these equations to obtain a nonlinear equation in one variable *z*. The solution can be found by graphical or trial and error methods without difficulty. We find that *z* = 0.050 yields an accurate solution with *x* = −707.2 which, in turn, yields
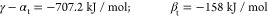
52

If we now return to the single diatomic π-bond, we see that this equation yields
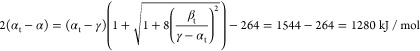
53

If we now assume that α = α_t_(*R* = ∞) = −1086 kJ/mol (the first ionization energy of the carbon atom), then we find the following tribasis HMO parameters
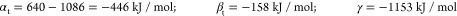
54

The ground state orbital energy for a diatomic π-bond is obtained as
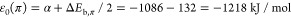
55

We note that the bridge function has an energy (−1153 kJ/mol) quite close to the ground state orbital energy (−1218 kJ/mol), while the antibonding excited state orbital energy is much higher (−446 kJ/mol). This tells us that the oscillation of the π-electrons between the atomic centers is very fast, and in fact, the electrons are predominantly in the spatial domain of the bridge function, i.e., between the two carbon nuclei, when the bond is at its normal length in the hydrocarbon molecules.

**Figure 13:**
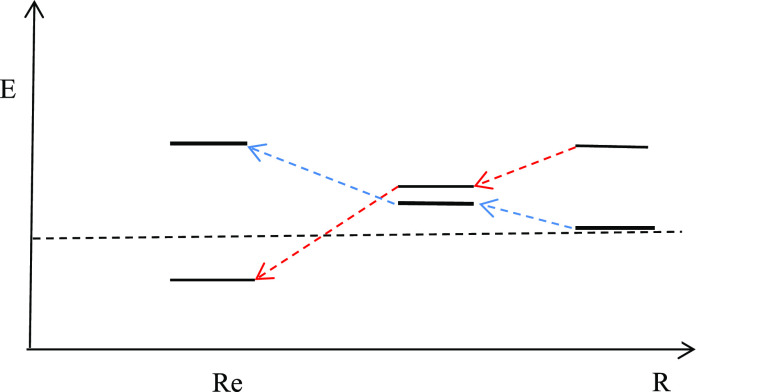
The energies of the degenerate local (α_t_, thick line, arrow up) and the bridge (γ, thin line, arrow down) basis functions switch as shown when the C–C bond length decreases from separate atom to molecular equilibrium value *R*_e_. The dashed line shows C 2p orbital energy.

It is clear that as the length *R* of the π-bond decreases from infinity, the ground state goes from being a bridged combination of local states to becoming predominantly a bridge state. The reason for this is, of course, that the local states are pushed away from the region between the nuclei, and their rise in energy is accompanied by a lowering of the bridge state energy. A graph showing the switch of position of the bridge (γ) and local state (α_t_) energies is found below in Figure 13.

This situation should not surprise. If covalent bonding is about electron sharing and transfer between bonded atoms, then the bridge state between the atoms might well be dominant at equilibrium bond length. The corresponding dynamical picture of the mechanistic change in the bonding as the molecule forms is noteworthy: at large *R*, the electrons hop infrequently between clearly recognized atomic states, but as *R* reaches the equilibrium value, the local states become strongly distorted, and the electrons move rapidly between two atomic centers. The interatomic motion has acquired an intrinsic two-center character at the equilibrium bond length. The motion between the centers is so fast that “hopping” or “flip–flop oscillation” are no longer apt descriptions. Instead, the rapid motion gives reality to the usual interpretation that the electrons are at both centers essentially simultaneously. From the point of view of energy analysis, one might also see this as an emergence of bond central character of the ground state as the localized states are pushed away from the region close to the nuclei by the ψ = 0 boundary condition at the bond mid-plane.

This empirical procedure of quantifying the analytical tHMO model is simplified and subject of improvement, but it does show that we can generate parameters such that the tribasis form of Hückel theory accounts for the bond energy and π → π* transition energy in ethylene and at the same time the aromaticity of benzene. Such a theory, which, in principle, is derivable directly from quantum chemistry with atomic orbital orthogonality accounted for, should allow delocalization phenomena to be explored not only in the usual planar aromatic hydrocarbon molecules but also in other molecular structures where further delocalization arises. From the point of view of mechanistic analysis of bonding, it is notable that when atomic orbital overlap is accounted for in terms of strictly local atomic and orthogonal bridge basis functions, then, as the molecule forms, the bridge goes from being a barrier to delocalization to becoming a trough dominating the shape of the wave function at small *R*. It appears that the equilibrium bond lengths of typical π-bonds are already “small *R*” in this sense.

### 4.4 Final Comments on the tHMO Model

As demonstrated for H_2_ above, the tribasis can be inserted in common methods of quantum chemistry to deal with many-electron features. Here, we have emphasized one-electron models, applying it to H_2_^+^ and venerable Hückel theory, in order to most simply demonstrate the insights gained by its semi-analytical and mechanistic modeling of electron delocalization, interatomic motion, and bonding. Hückel theory has allowed us to treat larger molecules and more than one electron without encountering the usual complications of interacting electrons. The responsible simplification of the tHMO, and of Hückel theory more generally, is the use of an effective one-electron potential which avoids the explicit treatment of electrostatic interactions and thereby also the dissociation error of Hartree–Fock theory. We should remember that the orbitals and energies obtained in this one-electron model are not the same as those of Hartree–Fock theory or DFT. These one-electron potentials are applicable for the neutral molecule, and they implicitly include electron–electron interaction and correlation. They reflect an assumption that the charge distribution seen by a single electron is locally atomic as if other electrons automatically move out of the way as the electron moves between atoms. This kind of perfect screening amounts to a correlation mechanism much like that of VB theory with only covalent configurations. Total electronic energies are obtained as sums of Hückel orbital energies, while for Hartree–Fock and DFT, the electrons are still treated as interacting, but their motion is independent and described by canonical orbital wave functions. In this latter case, the electron–electron repulsion must be evaluated explicitly, and the sum of orbital energies will lead to an overcounting of this repulsive energy by a factor of 2.

Another important simplification of Hückel theory and the tHMO model compared with rigorous quantum chemistry is the assumed site equality which allows us to generate parameters α_t_, β_t_, and γ for a reference diatomic structure and then keep these parameters for longer chain and ring structures. It is easy to see that this kind “uniform lattice structure” will not be found in a rigorous a priori methodology. An edge site will be different from an interior site, so structures with edges should in principle have site-to-site varying parameters. The uniform lattice format is most appropriate for fully site-symmetric structures like benzene. It is possible to refine the tHMO model to account for site inequivalence, but much of the simplicity will be lost, and we believe that it is worthwhile to explore the simplest form of the model first. The utility of the even more simplified original Hückel model is in this respect an encouragement.

The added strength of the tHMO model, as compared to its predecessor HMO model, is that the reference two-site system is treated with full account of atomic basis overlap before extrapolation into the form of a quantum lattice theory of delocalization. Therefore, the delocalization studied by the model is “dynamical delocalization”, i.e., the process of allowing strictly site-localized electrons to move between sites in the structure studied. In the original HMO model, one does not resolve the dramatic changes in the nature of sites and bridge states that we have found here. This is connected with the role of atomic basis overlap in interatomic electron motion. Hopefully, the mechanistic extension of the new tHMO model will help resolve more fully the connection between bonding and electron dynamics which has always been a focal point of Hückel theory.

## 5 Discussion and Conclusions

Why take the trouble to learn about and use a new, and somewhat awkward, tribasis method which improves the bond energies very marginally? The main reason is that this methodology allows us to extract significant new physical understanding from already existing and successful numerical analysis of chemical bonding in molecules. It enables us to explain in physical terms the most basic mechanism of covalent bonding. The simpler this mechanism can be explained and understood, and still account well for a more complex reality, the better. We claim that “dynamical delocalization”, i.e., the facilitated interatomic motion of valence electrons, is this most basic mechanism. It can be turned on and off in the tribasis numerical analysis. The delocalization mechanism, when defined this way, can be graphically illustrated extremely simply, e.g., for H_2_ as follows: H|H → H–H. The left image represents two hydrogen atoms, each with an electron confined to its own atomic half-space, and the right image is of two “bridged hydrogen atoms” with their electrons able to move, as freely as interactions allow, between the atomic subspaces.

The concept of “dynamical delocalization” is important for the understanding of covalent bonding since it clearly identifies the quantum mechanical stabilization associated with removal of constraint, in particular on the spatial motion of particles. The concept of delocalization is commonly used in energy decomposition analysis of bonding, but its dynamical implications are rarely considered. A major reason for this is that it is awkward to do so in mainstream quantum chemistry employing atomic basis functions which are not local. They penetrate into subspaces of neighboring atoms. Thus, it becomes difficult to assign physical meaning, e.g., in terms of interatomic electron motion and its rate, to a delocalization starting from the usual atomic basis functions. Therefore, we have shown here a way to define delocalization so that its dynamical implications are precise and clear, i.e., by defining it as the process of releasing electron motion in molecules from localization to separate atomic subspaces to delocalization over the full space of all atoms. Using this dynamical form of delocalization, we can clearly connect the standard energy analysis of bonding with its dynamical counterpart. We can pose the question whether covalent bonding can be predicted in reasonable agreement with experimental reality without including dynamical delocalization or, equivalently, interatomic electron motion. All the evidence suggests that this is not possible. We are then forced to conclude that covalent bonding is most easily explained by a revision of the usual simple models of bonding to account for the role of interatomic electron dynamics. The “electron pair sharing” mechanism of the Lewis model can be retained, but rather than sitting still at bond midpoints, these paired electrons should be understood to oscillate in a more or less correlated way between the two atomic centers so as to keep them neutral rather than charged. The correlation in this paired electron motion can be expected to be strongest (in the sense of most needed) in the long bond length limit but decrease for smaller *R*, and around equilibrium, one can expect the electron motion between the atomic centers to be predominantly independent and uncorrelated. This suggests that we should reinterpret the still very important Lewis “electron dot picture” of bonding to include the Feynman “flip–flop” mechanism of covalent bonding with the understanding that the electrons jump synchronously in opposite directions for large *R* but move nearly independently at *R*_e_ and so fast that “jump” or “flip–flop” may not be the right words to use. This has been made clear in the Hückel analysis but can be seen also in the results for H_2_ obtained by the correlated VB method.

The view of covalent bonding as a dynamical mechanism has many other dividends in terms of physical understanding of electronic structure theory. We have pointed out that it explains why Thomas–Fermi theory failed to account for covalent bonding, i.e., by neglecting dynamical constraints on electron dynamics and thereby failing to resolve the gradual onset of delocalization dynamics as the molecule forms.^20^ In turn, this explains why the promise of the Hohenberg–Kohn theorem^41^ of true DFT has not been fulfilled; i.e., we do not know how to include dynamical processes such as covalent bonding in “density-only quantum mechanics”. The Kohn–Sham scheme^42^ reintroduced quantum kinetic energy and one-electron orbitals which account for gradual delocalization and onset of interatomic electron motion.^24,43^ Thus, in this dynamical perspective, modern DFT is successful because it can resolve the quantum mechanical coupling between energy and dynamics which is absent in classical mechanics.

The tribasis approach not only emphasizes the role of dynamical delocalization in bonding but also casts light on the repulsion associated with localization. We can call it a Pauli repulsion since it leads to satisfaction of the Pauli principle by electron separation. It can also be referred to as a steric repulsion. It is the possibility of resolving the combination of this repulsion with delocalization that makes it attractive to use the tribasis Hamiltonian structure to refine Hückel theory of electron delocalization. The original Hückel model of π-electron delocalization in planar aromatic hydrocarbon molecules has long provided a view of covalent bonding which relates it to electron motion in chains or rings of sites in the form of carbon atoms. It has also been able to explain the extra stability of aromatic molecules with further delocalization, i.e., beyond that achieved by local pair bonds, but it has never been able to account for this combination of repulsive overlap and more strongly attractive dynamical delocalization effects. This balancing of overlap repulsion and the delocalization attraction thereby afforded is, of course, always present in accurate quantum chemical methods, if not always the focal point of interpretation.^44^ The tHMO model introduced here includes explicit account for these two important mechanisms in the parameterized form. It remains to be tested in widespread applications, but it should, in principle, be able to resolve both spectroscopic transition energies and bond energies in planar π-electron structures with a single set of parameters. At least, we have now included in the model the competing mechanisms of delocalization and steric repulsion which act differently on these two types of energies.

The tribasis analysis introduced here is capable of much extension and generalization. Some have already been mentioned above. Larger homogeneous diatomic molecules could be studied in much the same way as H_2_ mentioned above. Hydrogenic molecules like H_3_, H_4_, H_6_, etc., could be studied by a slightly generalized tHMO model. The method can work with larger basis sets divisible into local and bridge subgroups. The potentials can be more general. In the longer term, it may be extended to asymmetric bonds A–B where A and B are different atoms. In such bonds, the reliable identification of separate contributions from covalent and ionic mechanisms would be an important goal. For larger molecules with nonbonding electrons, the separation of the total bond energy into delocalization and Pauli repulsion energy becomes more complex but, we predict, still accessible by the tribasis methodology. If delocalization is a bonding mechanism, its opposite, localization, is not just nonbonding but antibonding due to the Pauli repulsion associated with it. For H_2_, the delocalization attraction is dominant, but for He_2_, we expect to see a dominant localization repulsion. Double and triple bonds should also be considered and hybridized atomic basis functions. The systematic method, used in the tribasis approach, of generating orthogonal local and bridge basis functions, also offers opportunities to revisit the long-standing problem of implementation of VB theory for larger molecules.
